# A mitochondria-targeted caffeic acid derivative reverts cellular and mitochondrial defects in human skin fibroblasts from male sporadic Parkinson's disease patients

**DOI:** 10.1016/j.redox.2021.102037

**Published:** 2021-06-08

**Authors:** Cláudia M. Deus, Susana P. Pereira, Teresa Cunha-Oliveira, José Teixeira, Rui F. Simões, Fernando Cagide, Sofia Benfeito, Fernanda Borges, Nuno Raimundo, Paulo J. Oliveira

**Affiliations:** a PhD Programme in Experimental Biology and Biomedicine (PDBEB), Institute for Interdisciplinary Research (IIIUC), University of Coimbra, Coimbra, Portugal; b CNC-Center for Neuroscience and Cell Biology, CIBB - Centre for Innovative Biomedicine and Biotechnology, University of Coimbra, Coimbra, Portugal; c Research Centre in Physical Activity Health and Leisure (CIAFEL), Faculty of Sports, University of Porto, Porto, Portugal; d CIQUP/Department of Chemistry and Biochemistry, Faculty of Sciences, University of Porto, Porto, Portugal; e Penn State University College of Medicine, Department of Cellular and Molecular Physiology, Hershey, PA, USA; f Multidisciplinary Institute of Ageing (MIA), University of Coimbra, Coimbra, Portugal

**Keywords:** Human skin fibroblasts, Metabolism, Mitochondria, Mitochondriotropic antioxidant, Sporadic Parkinson's disease

## Abstract

Parkinson's Disease (PD) is a neurodegenerative disorder affecting more than 10 million people worldwide. Currently, PD has no cure and no early diagnostics methods exist. Mitochondrial dysfunction is presented in the early stages of PD, and it is considered an important pathophysiology component. We have previously developed mitochondria-targeted hydroxycinnamic acid derivatives, presenting antioxidant and iron-chelating properties, and preventing oxidative stress in several biological models of disease. We have also demonstrated that skin fibroblasts from male sporadic PD patients (sPD) presented cellular and mitochondrial alterations, including increased oxidative stress, hyperpolarized and elongated mitochondria and decreased respiration and ATP levels. We also showed that forcing mitochondrial oxidative phosphorylation (OXPHOS) in sPD fibroblasts uncovers metabolic defects that were otherwise hidden. In this work, we tested the hypothesis that a lead mitochondria-targeted hydroxycinnamic acid derivative would revert the phenotype found in skin fibroblasts from sPD patients.

Our results demonstrated that treating human skin fibroblasts from sPD patients with non-toxic concentrations of AntiOxCIN_4_ restored mitochondrial membrane potential and mitochondrial fission, decreased autophagic flux, and enhanced cellular responses to stress by improving the cellular redox state and decreasing reactive oxygen species (ROS) levels. Besides, fibroblasts from sPD patients treated with AntiOxCIN_4_ showed increased maximal respiration and metabolic activity, converting sPD fibroblasts physiologically more similar to their sex- and age-matched healthy controls. The positive compound effect was reinforced using a supervised machine learning model, confirming that AntiOxCIN_4_ treatment converted treated fibroblasts from sPD patients closer to the phenotype of control fibroblasts.

Our data points out a possible mechanism of AntiOxCIN_4_ action contributing to a deeper understanding of how the use of mitochondria-targeted antioxidants based on a polyphenol scaffold can be used as potential drug candidates for delaying PD progression, validating the use of fibroblasts from sPD patients with more active OXPHOS as platforms for mitochondria-based drug development.

## 1 Introduction

Parkinson's Disease (PD) is a neurodegenerative disorder affecting more than 10 million people worldwide [1]. Currently, PD is an incurable disease, with available drugs only alleviating symptoms. However, in more advanced stages of the disease, clinical treatment is no longer effective in controlling PD symptoms [2]. Parkinson's Disease is characterized by aggregation of α-synuclein, ubiquitin, neurofilaments, and molecular chaperones, which are present as Lewy Bodies (LBs) [3], culminating in dopaminergic cell neurodegeneration [4]. Only a small proportion of the PD cases results from genetic mutations, while around 85–90% of the cases are sporadic [5]. The etiology of sporadic PD is not fully understood, which also results from the low number of experimental models used, as well as the experimental difficulty in obtaining suitable human tissues to study PD pathogenesis [5].

Mitochondrial dysfunction and oxidative stress have been extensively implicated in PD pathogenesis [[6], [7], [8], [9]]. Mitochondrial dysfunction in PD leads to increased oxidative stress and/or vice versa [[10], [11], [12]]. Both mitochondrial dysfunction and oxidative stress are present in the early stages of the disease, even before motor symptoms appear [13,14]. Besides, PD is considered a multisystem disorder [15,16], in which mitochondrial dysfunction and oxidative stress are not only limited to the brain but extend to peripheral tissues, namely gastrointestinal tract, salivary glands, skin, retina, heart, and others [17]. It has been reported that peripheral mitochondrial dysfunction correlates with clinical severity in sporadic PD [18]. Peripheral cells, including fibroblasts, are a robust and available tool and reflect cumulative aging-related cell damage. Fibroblasts can be reprogrammed to induced-pluripotent cells and differentiated into dopaminergic neurons [[18], [19], [20]]. However, they also are a proxy model of metabolic and mitochondrial dysfunction in PD, even before motor symptoms manifestation. It is thought that toxic metabolites produced in other parts of the body can likely activate inflammatory mechanisms in the central nervous system (CNS) after being transported through the bloodstream and crossing the blood-brain barrier (BBB) into the brain, promoting neuroinflammation [21]. Nonetheless, we should note that using fibroblasts from sPD patients has limitations, as previously described [[22], [23], [24], [25]]. However, given the scarcity of models for sporadic PD, the advantages of using donor fibroblasts as a model outweigh their limitations [23,26].

Alterations in antioxidant defense mechanisms have been related to PD pathogenesis [27,28]. In fact, altered activity of several antioxidant enzymes has been demonstrated in PD models; remarkably, catalase activity was reduced in *substantia nigra* and *putamen* of PD brains [27,29]. On the other hand, peroxiredoxin 1 (PRx1), PRx2, and PRx4 overexpression protected against 6-hydroxydopamine (6-OHDA)-induced dopaminergic cell death, while the silencing of mitochondrial PRx3 and PRx5 increased sensitivity to 1-methyl-4-phenylpyridinium (MPP^+^) [27,30]. Furthermore, decreased levels of glutathione (GSH) are one of the earliest biochemical alterations associated with PD, due to GSH loss which occurs in incidental LBs disease, which is considered an asymptomatic precursor of PD [31]. Accordingly, PD brains showed no changes in superoxide dismutase 1 (SOD1) activity, while superoxide dismutase 2 (SOD2) activity was increased [32], suggesting that SOD2 is highly inducible in response to increased oxidative stress. In contrast, both catalase and glutathione peroxidase activities were decreased in PD brains [33]. Alterations in the antioxidant defense system have already been described in peripheric tissues [34,35]. Thus, targeting mitochondrial oxidative stress with antioxidants that can specifically reduce over-stimulated mitochondrial ROS production in pathological processes may play a vital role in delaying disease pathogenesis and progression. Some mitochondria-targeted antioxidants have been tested in PD models, showing beneficial effects, such as MitoQ, MitoVitE, MitoTEMPO, MitoApocynin, MitoPBN and MitoSOD [36]. Although attractive, these strategies' success has been hampered by several challenges and limitations, with no approach having yet resulted in approved drugs for PD therapy. So, new chemical entities directed to mitochondria have been generated to overcome the putative pitfalls of clinical assays.

Hydroxycinnamic acids (HCAs) are the major subgroup of phenolic acids ubiquitously distributed in plants, including tea leaves, coffee, red wine, various fruits (especially red-colored ones), vegetables and whole grains. Several studies have demonstrated HCAs' effectiveness for preventive and/or therapeutic purposes in several oxidative stress-related diseases, such as atherosclerosis, inflammatory injury, cancer, and cardiovascular diseases [37]. Their antioxidant-related mechanism of action was initially suggested to depend on the radical-scavenging activity. However, other mechanisms of action are more likely to occur *in vivo*, including inhibition of ROS- and reactive nitrogen species (RNS)-generating enzymes, modulation of gene expression through ARE/Nrf-2 pathway, and chelation of transition metals, such as copper or iron [38,39]. A mitochondriotropic antioxidant based on natural dietary caffeic acid, AntiOxCIN_4_, was developed [40]. AntiOxCIN_4_ showed remarkable antioxidant and iron-chelation properties and inhibited oxidative damage either in isolated liver mitochondria or human HepG2 cells [40]. We have recently demonstrated in primary human skin fibroblasts that AntiOxCIN_4_ increases ROS generation, leading to up-regulation of NRF2 gene expression and activation of antioxidant pathways [41]. Furthermore, neuroprotective effects were also demonstrated in human SH-SY5Y cells against 6-OHDA-induced oxidative damage [42]. Additionally, AntiOxCIN_4_ can play a role in maintaining intracellular GSH homeostasis by increasing its supply [40].

We recently demonstrated that fibroblasts from sPD patients show hyperpolarized and elongated mitochondrial networks and higher mitochondrial ROS concentration, as well as decreased adenosine triphosphate (ATP) levels, glycolysis-related extracellular acidification rate (ECAR) and oxygen consumption rate (OCR). Moreover, we also showed that forcing mitochondrial oxidative phosphorylation (OXPHOS) uncovers metabolic defects that were otherwise hidden in the same cell type [43]. In this context, the present work hypothesized that AntiOxCIN_4_ reverts the *in vitro* phenotype found in skin fibroblasts from sPD patients. We confirmed our hypothesis showing that AntiOxCIN_4_ can be a valuable drug candidate in the context of PD.

## 2 Materials and methods

### 2.1 Reagents

Bovine serum albumin (BSA), ammonium persulfate (APS), Bradford reagent, brilliant blue G, calcium chloride (CaCl_2_), dimethyl sulfoxide (DMSO), ethylenediaminetetraacetic acid (EDTA), glycerol, glycine, β-mercaptoethanol 98%, phenylmethylsulfonyl fluoride (PMSF), sodium chloride (NaCl), sodium dodecyl sulfate (SDS), sulforhodamine B sodium salt (SRB), trizma base, Tris pH 8.8, Tris pH 6.8 and trypan-blue solution were obtained from Sigma-Aldrich (Saint Louis, MO, USA). Acetic acid, ethanol, hydrochloric acid (HCl), magnesium chloride (MgCl_2_), methanol, potassium chloride (KCl), potassium phosphate monobasic (KH_2_PO_4_), sodium hydrogencarbonate (NaHCO_3_), sodium sulfate (NaSO_4_) and sodium hydroxide (NaOH) were also obtained from Merck (Whitehouse Station, NJ, USA). Acrylamide, Laemmli buffer, PVDF membranes, ECL detection system and N,N,N′,N′-Tetramethylethylenediamine (TEMED) were obtained from BioRad (Hercules, CA, USA). The fluorescent dyes tetramethyl rhodaminemethylester (TMRM), Hoechst 33,342 and Lysotracker red were obtained from Molecular Probes (Eugene, OR, USA). Dulbecco's modified Eagle's medium without glucose (DMEM, 5030), Earle's Balanced Salt Solution (EBSS, 14155063), penicillin, streptomycin, fetal bovine serum (FBS), and 0.25% Trypsin–EDTA were purchased from Gibco-Invitrogen (Grand Island, NY). Cell lysis buffer (9803) was obtained from Cell Signaling Technology (Danvers, MA, USA).

All reagents and chemical compounds used were of the largest degree of purity commercially available. In the preparation of every solution, ultrapure distilled water (conductivity < 18 μS.cm^−1^), filtered by a Milli Q Millipore system, was used to minimize as much as possible contamination with metal ions.

### 2.2 Synthesis of AntiOxCIN_4_

The mitochondriotropic compound AntiOxCIN_4_ was synthetized as described by Ref. [40].

### 2.3 Cell culture conditions

Skin fibroblasts from five sporadic late-onset PD (sPD) male patients and five age- and sex-matched healthy controls were obtained from a cell line repository of the Coriell Institute for Medical Research, USA (www.coriell.org), and their detailed information was previously described [43].

Fibroblasts from sPD patients were cultured in DMEM (D5030) supplemented with 0.9 g/L glucose, 1.8 g/L sodium bicarbonate, 0.584 g/L glutamine, 0.11 g/L sodium pyruvate, 100 U/ml of penicillin, 100 g/ml of streptomycin, and 10% fetal bovine serum in sterile tissue-culture dishes at 37 °C in a humidified atmosphere of 5% CO_2_. Cell culture medium was changed in the last 24 h of the experiments to a Dulbecco's Modified Eagle's Medium (DMEM, 5030) without glucose and supplemented with 1.8 g/L sodium bicarbonate, 0.11 g/L sodium pyruvate, 1.8 g/L galactose, 0.584 g/L l-glutamine, 100 U/ml of penicillin, 100 g/ml of streptomycin, and 10% fetal bovine serum with the same culture conditions described before. This culture medium was used to force cells to rely mainly on OXPHOS for ATP production [[43], [44], [45]] and is described here as OXPHOS medium (OXPHOSm). Cells were passaged by trypsinization using standard methods when reaching 70–80% confluence, and all experiments were prepared from cultures in log-phase growth.

**Fig. 1:**
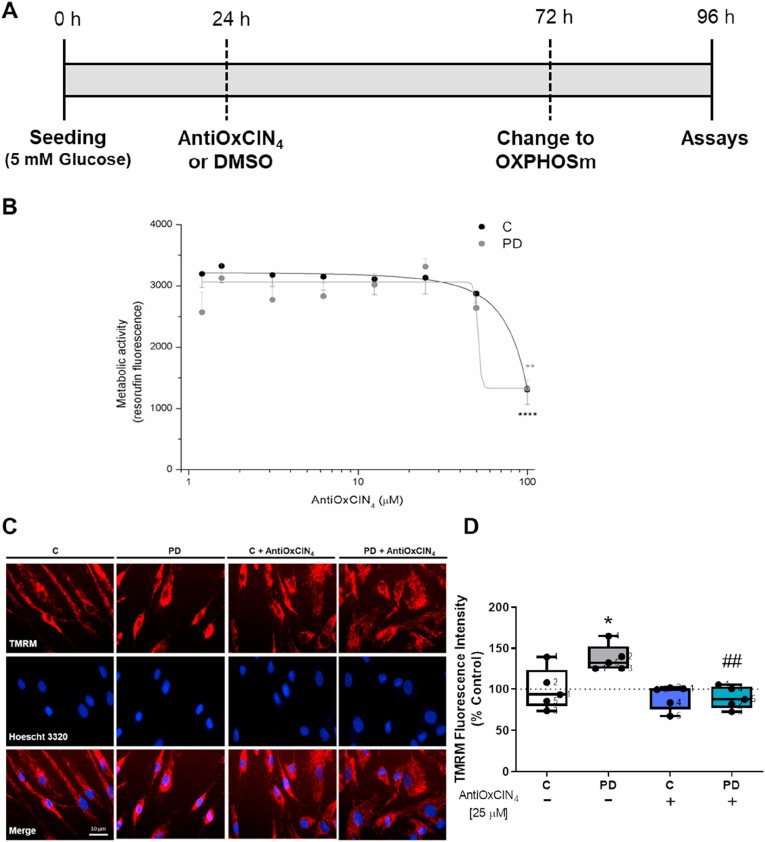
(A) Experimental design; (B) AntiOxCIN_4_ cytotoxicity measured by resazurin assay; (C) Effects of AntiOxCIN_4_ on mitochondrial polarization of human skin fibroblasts from sPD patients (PD) and their sex- and age-matched controls. The quantification of TMRM fluorescence was obtained by ImageJ 1.45S program. Data are expressed as mean ± SEM of TMRM intensity fluorescence divided by area (D). Typical image of fibroblasts from sPD patients labeled with mitochondrial Δψ–dependent fluorescent probe TMRM (E). Mitochondrial network elongation (F), interconnectivity (G), and swelling (H) of these TMRM fluorescent cells were measured using an ImageJ macro (n = 45 cells per condition). Western blotting was used to detect phosphorylation of dynamin-related protein 1 at serine 637 (p-DRP1 ser637) and mitochondrial fission 1 (FIS1) (I and J) protein contents in total fractions from human skin fibroblasts cell lines. Actin was used as a loading control. Blots are representative of different cell preparations with a random distribution between C and PD. Each measurement corresponds to one different individual (5 fibroblasts from sPD patients and 5 fibroblasts from respective sex- and age-matched healthy controls). Data was normalized by the control condition (C = 100% or C = 1.0 fold-change). Data are expressed as mean ± SEM of 5 different experiments. Statistical significance was accepted with (*) p < 0.05 to C vs PD or C + AntiOxCIN_4_ vs PD + AntiOxCIN_4_, (**) p < 0.01 to C vs PD or C + AntiOxCIN_4_ vs PD + AntiOxCIN_4_, (^#^) p < 0.05 to C vs C + AntiOxCIN_4_ or PD vs PD + AntiOxCIN_4_, (^##^) p < 0.01 to C vs C + AntiOxCIN_4_ or PD vs PD + AntiOxCIN_4_.

Considering that cell passage number can affect cell phenotype and responses, all experiments were performed with cells from passage 6 to 15, and cell passage was maintained between fibroblasts from sPD patients and their age- and sex-matched controls. A stock solution of 100 μM of AntiOxCIN_4_ was prepared in dimethylsulfoxide (DMSO) and stored frozen. Vehicle controls received an equivalent amount of DMSO, which never exceeded 0.1% v/v. All experiments were performed according to the scheme of Fig. 1A. AntiOxCIN_4_ or DMSO treatment was only performed once (24 h after seeding).

### 2.4 Cell viability measurements

The resazurin assay was used to measure the cellular viability based on living cells' metabolic activity through the bioreduction of the dye from the oxidized form (blue) to the fluorescence product (red) by fluorescence. For AntiOxCIN_4_ cytotoxicity assays, cells were seeded at a concentration of 15,000 cells/cm^2^ in 96-well plates, with a final volume of 100 μl per well and allowed to proliferate for 24 h. Then, 1.5, 3, 6.25, 12.5, 25, 50 and 100 μM of AntiOxiCIN_4_ was added to cells during 48 h. In the last 24 h of the experiment, cell culture medium was changed for OXPHOSm. Resazurin stock solution (1 mg/ml) was prepared in PBS 1x and stored at −20 °C. Afterward, cell medium from each well was removed, and cells were washed with PBS 1x. After removing PBS 1x, 100 μl of resazurin solution (1:1000 dilution in growth medium from a stock solution) was added in each well and incubated for 5 h at 37 °C with a 5% CO_2_ atmosphere. Resazurin is irreversibly converted into resorufin, whose fluorescence was measured in a Biotek Cytation 3 spectrophotometer (BioTek Instruments Inc., USA) at 540/590 nm [46].

### 2.5 Cell proliferation measurements

The sulforhodamine B (SRB) assay was used to measure cellular proliferation. Cells were seeded at a concentration of 15,000 cells/cm^2^ in 96-well plates, with a final volume of 100 μl per well and allowed to proliferate for 24 h. Then, 25 μM of AntiOxCIN_4_ was added to cells for 48 h. In the last 24 h of the experiment, cell culture medium was changed for OXPHOSm. Afterward, the incubation medium was removed, and cells were fixed in 1% acetic acid in ice-cold methanol for at least one day at – 20 °C. Cells were then incubated with 0.05% (w/v) SRB reagent dissolved in 1% acetic acid for 1 h at 37 °C. Unbound dye was removed with 1% acetic acid. Dye bound to cell proteins was extracted with 10 mM Tris-base solution, pH 10. After SRB labeling, absorbance was measured in a Biotek Cytation 3 spectrophotometer (BioTek Instruments Inc., USA) at 510 nm, and the amount of dye released is proportional to the number of cells in each well [46,47].

### 2.6 Adenosine triphosphate (ATP) level measurements

Intracellular ATP levels were measured by using CellTiter-Glo Luminescent Cell Viability Assay (G7571, Promega) following the manufacturer's instructions. Cells were seeded at a concentration of 15,000 cells/cm^2^ in white opaque-bottom, 96-well plates, with a final volume of 100 μl per well and allowed to proliferate for 24 h. Then, 25 μM of AntiOxCIN_4_ was added to cells for 48 h. In the last 24 h of the experiment, cell culture medium was changed for OXPHOSm. Afterward, 50 μL of cell culture medium was removed and 50 μL CellTiter-Glo® Reagent (CellTiter-Glo Buffer + CellTiter-Glo Substrate) was added to the cells. Contents were mixed for 2 min on an orbital shaker to induce cell lysis and, after 10 min of incubation at 22 °C, the luminescence signal was monitored in a Biotek Cytation 3 spectrophotometer (BioTek Instruments Inc., USA). ATP standard curve was also generated following the manufacturer's instructions. The luminescence signal was proportional to the amount of ATP present in the solution [48].

### 2.7 Mitochondrial network characterization and determination of reactive oxygen species by vital epifluorescence microscopy

Vital epifluorescence microscopy was used to detect alterations in mitochondrial polarization and network distribution in human skin fibroblasts from sPD patients by using the mitochondrial membrane potential-dependent (Δψmt) dye TMRM, as well as to assess oxidative stress by using CM-H_2_-DCFDA dye and Hoescht 33,342 to stain DNA. Cells were seeded at a concentration of 7500 cells/cm^2^ in black clear-bottom 24-well plates, with a final volume of 500 μl per well and allowed to proliferate for 24 h. Then, 25 μM of AntiOxCIN_4_ was added to cells during 48 h. In the last 24 h of the experiment, the cell culture medium was replaced for OXPHOSm. Afterward, fluorescent dyes, TMRM (100 nM), CM-H_2_-DCFDA (3 μM) and Hoescht 33,324 (1 mg/mL) in microscopy solution buffer (120 mM NaCl, 3.5 mM KCl, 0.4 mM NaH_2_PO_4_, 20 mM HEPES, 5 mM NaHCO_3_, 1.2 mM Na_2_SO_4,_ and 10 mM sodium pyruvate) and supplemented with 1.2 mM MgCl_2_ and 1.3 mM CaCl_2_ were added in each well and incubated for 15–30 min at 37 °C with a 5% CO_2_ atmosphere. Images were obtained using the 40× objective of the In Cell Analyzer 2200 (GE, Healthcare) microscope and were treated with ImageJ 1.45S program.

### 2.8 Determination of mitochondrial morphology

In order to quantify mitochondrial morphology, a previously validated Image J macro was used [49]. Pictures of human skin fibroblasts from sPD patients stained with TMRM were converted into grayscale, inverted to show mitochondria-specific fluorescence as black pixels, and a threshold was applied to optimally resolve individual mitochondria. The mean area/perimeter ratio was employed as an index of mitochondrial interconnectivity, inverse circularity was used as a measure of mitochondrial network elongation, and mean area/perimeter ratio normalized to circularity was used to measure mitochondrial swelling.

### 2.9 Vital confocal fluorescence microscopy

Vital confocal fluorescence microscopy was used to detect the presence of acidic bodies (lysosomes) in human skin fibroblasts from sPD patients by using Lysotracker Red. Cells were seeded at a concentration of 7500 cells/cm^2^ in 8-well chamber coverslip, with a final volume of 300 μl per well and allowed to proliferate for 24 h. Then, 25 μM of AntiOxCIN_4_ was added to cells for 48 h. In the last 24 h of the experiment, the cell culture medium was changed for OXPHOSm. Afterward, a fluorescent dye, Lysotracker Red (75 nM) in microscopy solution buffer (120 mM NaCl, 3.5 mM KCl, 0.4 mM NaH_2_PO_4_, 20 mM HEPES, 5 mM NaHCO_3_, 1.2 mM Na_2_SO_4,_ and 10 mM sodium pyruvate) was added in each well and incubated for 30 min at 37 °C with a 5% CO_2_ atmosphere. 2 μM trichostatin during 24 h, and 500 nM bafilomycin during 4 h were used as a positive and negative control, respectively. Images were obtained using the 40× objective of the laser scanning confocal microscope (LSM 710, Zeiss) with environmental control chamber. Quantification of Lysotracker Red fluorescence intensity was obtained with FIJI/ImageJ 1.52p program.

### 2.10 Determination of intracellular oxidative stress

To evaluate intracellular oxidative stress, we used the CM-H_2_-DCFDA fluorescent dye. Cells were seeded at a concentration of 15,000 cells/cm^2^ in 96-well plates, with a final volume of 100 μl per well and allowed to proliferate for 24 h. Then, 25 μM of AntiOxCIN_4_ was added to cells for 48 h. In the last 24 h of the experiment, the cell culture medium was changed for OXPHOSm. Afterward, CM-H_2_-DCFDA (3 μM) in a microscopy solution buffer (120 mM NaCl, 3.5 mM KCl, 0.4 mM NaH_2_PO_4_, 20 mM HEPES, 5 mM NaHCO_3_, 1.2 mM Na_2_SO_4,_ and 10 mM sodium pyruvate) supplemented with 1.2 mM MgCl_2_ and 1.3 mM CaCl_2_ were added in each well and incubated for 20 min at 37 °C with a 5% CO_2_ atmosphere. The fluorescence was measured every 2 min for 90 min in a Biotek Cytation 3 spectrophotometer (BioTek Instruments Inc., USA) at 485/530 nm. At the end of the experiment, the incubation media were removed, and cells were fixed in 1% acetic acid in ice-cold methanol for at least one day at – 20 °C. Sulforhodamine B assay was used to evaluate cell mass as described above and to normalize results.

### 2.11 Measurement of total superoxide dismutase (SOD) activity

Total SOD activity was measured by using a SOD activity assay kit (SOD activity Enzo Life Sciences, USA) following the manufacturer's instructions. Briefly, cells were seeded at a concentration of 15,000 cells/cm^2^ in tissue-culture dishes, with a final volume of 10 ml and allowed to proliferate for 24 h. Then, 25 μM of AntiOxCIN_4_ was added to cells for 48 h. In the last 24 h of the experiment, the cell culture medium was changed for OXPHOSm. Afterward, cells were harvested, washed with ice-cold 1x PBS, and lysed as described in kit protocol. The protein concentration was determined using Bradford assay, using bovine serum albumin (BSA) as a standard [50]. Cell lysates of each sample or standard (25 μL) were incubated with 150 μL reaction mixture containing WST-1 and xanthine oxidase and then xanthine solution was added. Formazan formation was measured for 10 min at 37 °C, and the absorbance was monitored at 450 nm in a Biotek Cytation 3 spectrophotometer (BioTek Instruments Inc., USA). SOD standard curve was also generated following the manufacturer's instructions.

### 2.12 Measurement of reduced and oxidized glutathione levels

Reduced (GSH), oxidized (GSSG) and total cellular GSH/GSSG ratio levels [51] were determined by a GSH/GSSG-Glo assay kit (V6612, Promega, USA) following the manufacturer's instructions. Briefly, cells were seeded at a concentration of 15,000 cells/cm^2^ in white opaque-bottom, 96-well plates, with a final volume of 100 μl per well and allowed to proliferate for 24 h. Then, 25 μM of AntiOxCIN_4_ was added to cells for 48 h. In the last 24 h of the experiment, the cell culture medium was changed to OXPHOSm. Afterward, cell culture medium was removed, and 50 μL of total glutathione or oxidized glutathione lysis reagents were added and were mixed for 5 min on an orbital shaker to induce cell lysis. After, 50 μL of luciferin generation reagent was added and incubated at 22 °C for 30 min. Lastly, 100 μL of luciferin detection reagent was added, and after 15 min of incubation at 22 °C, the luminescence signal was monitored in a Biotek Cytation 3 spectrophotometer (BioTek Instruments Inc., USA). A glutathione standard curve was also generated following the manufacturer's instructions.

### 2.13 Protein semi-quantitation by Western blotting

Cells were seeded at a concentration of 15,000 cells/cm^2^ in tissue-culture dishes, with a final volume of 10 ml and allowed to proliferate for 24 h. Then, 25 μM of AntiOxiCIN_4_ was added to cells for 48 h. In the last 24 h of the experiment, cell culture medium was changed for OXPHOSm. Afterward, to obtain total cellular extracts, cells were harvested by trypsinization and washed once with PBS 1x. To collect the cells, two centrifugation steps were performed for 5 min at 1000×*g* (4 °C). The cellular pellet was resuspended in cell lysis buffer 1x (Bio-Rad, 9803) supplemented with 100 μM phenylmethylsulfonyl fluoride (PMSF). The protein concentration in cellular extracts was determined by the Bradford assay, using BSA as a standard [50]. Samples were sonicated, and 25 μg of total protein extract was separated by electrophoresis in a 12% SDS-PAGE gel, after denaturation at 95 °C for 5 min in Laemmli buffer (Bio-Rad) and electrophoretically transferred to 0.45 μm PVDF membranes. After blocking with 5% milk in TBST (50 mM Tris–HCl, pH 8; 154 mM NaCl and 0.1% tween 20) for 2 h at room temperature, PVDF membranes were incubated overnight at 4 °C under constant stirring with primary antibodies against FIS1 (1:1,000, sc-376,447, Santa Cruz), p-DRP1 (ser 637) (1:1,000, 6319s, Cell Signaling), SIRT1 (1:1,000, ab110304, Abcam), SIRT3 (1:1,000, 5490, Cell Signaling), BAX (1:500, 2772, Cell Signaling), SOD2 (1:1,000, ab13533, Abcam), LC3 (1:1,000, 3868, Cell Signaling), TOM20 (1:1,000, sc11415, Santa Cruz), Total OXPHOS cocktail (1:1,000, ab110411, Abcam), p66^shc^ (1:1,000, 610,879, BD Transduction Laboratories), p-p66^shc^ (ser36) (1:1,000, 566,807, Calbiochem), p62 (1:1,000, PM045, MBL), AMPK-α (1:1,000, 2603, Cell Signaling), p-AMPK-α (thr 172) (1:1,000, 2531, Cell Signaling) PGC1-α (1:750, sc13067, Santa Cruz) and actin (1:5,000, MAB1501, Millipore). Membranes were washed three times with TBST for 5 min and incubated with the correspondent horseradish peroxidase-conjugated secondary antibody, goat anti-mouse IgG (1:2,500, Santa Cruz), and goat anti-rabbit IgG (1:2,500, Santa Cruz) for 1 h at room temperature. Membranes were then incubated with the Clarity Western ECL Substrate (170–5061, Bio-Rad) and imaged with the VWR Gel Imaging system (VWR, Portugal). Each band's densities were calculated with ImageJ 1.45S program and normalized for the respective actin band density.

### 2.14 Cellular oxygen consumption rate measurements

Oxygen consumption rate (OCR) and extracellular acidification rate (ECAR) were measured at 37 °C using a Seahorse XF^e^96 Extracellular Flux Analyzer (Agilent Technologies, Germany) [52]. The test performed was the Seahorse XF Cell Mito Stress (Agilent Technologies, Santa Clara, CA). Cell lines were seeded under the same conditions described above at a density of 70,000 cells/cm^2^. A XF^e^96 sensor cartridge for each cell plate was placed in a 96-well calibration plate containing 200 μL/well calibration buffer and left to hydrate overnight at 37 °C. The cell culture medium from the plates was replaced the following day with 180 μL/well of pre-warmed, low-buffered, serum-free minimal DMEM (102,353, Bioscience) medium, with the pH adjusted to 7.4 and incubated at 37 °C for 1 h to allow the temperature and pH of the medium to reach equilibrium before the first-rate measurement. Oligomycin, FCCP, rotenone, and antimycin A were prepared in DMSO. Three μM oligomycin, 2 μM FCCP, and 2.5 μM rotenone plus 2.5 μM antimycin A were injected into reagent delivery port A, B, and C, respectively. Next, 20, 22, and 24 μL of compounds were pre-loaded into the ports, respectively, of each well in the XF^e^96 sensor cartridge. Three baseline rate measurements of OCR were made using a 3 min mix, 5 min measure cycle. The compounds were then pneumatically injected by the XF^e^96 Analyzer into each well, mixed, and measurements of OCR made using a 3 min mix, 5 min measure cycle. In the end of the experiment, cells were fixed by adding 50 μL of 60% trichloroacetic acid and stored for at least one day at 4 °C. Sulforhodamine B assay was used to evaluate cell mass as described above to normalize results. Results were analyzed by using the Software Wave Desktop Version 2.2.

### 2.15 Analysis of mitochondrial DNA (mtDNA) copy number

Cells were collected and centrifuged at 2000×*g* for 5 min. The pellets were washed in 5 mL of PBS, and the suspension was centrifuged at 2000×*g* for 5 min. The resulting pellets were stored at −80 °C until DNA extraction. Total DNA was extracted from cell pellets using the QIAamp DNA mini kit (Qiagen, Düsseldorf, Germany), following manufacturer's protocols, sonicated in a water bath for 10 min, and quantified using a Nanodrop2000 (ThermoScientific, Waltham, MA, USA). RT-PCR was performed using the SsoFast Eva Green Supermix, in a CFX96 real time-PCR system (Bio-Rad, Hercules, CA, USA), with the primers defined in Table 1, at 500 nM. Amplification of 25 ng DNA was performed with an initial cycle of 2 min at 98 °C, followed by 40 cycles of 5 s at 98 °C and 5 s at 60 °C. The amplification specificity was assessed at the end of the amplification by a melting curve between 65 and 95 °C, using an increment of 0.5 °C in each step. At the end of each cycle, Eva Green fluorescence was recorded to enable the determination of Cq. For each set of primers, amplification efficiency was assessed, and no template controls were run. mtDNA copy number was determined in each sample by the ratio between the amount of a fragment of the mitochondrial cytochrome B (mito CytB) and the amount of the beta-2-microglobulin (B2m) nuclear gene, using the CFX96 Manager software (v. 3.0; Bio-Rad).

**Table 1:** Sequences of primers used for the analysis of mitochondrial DNA copy number and gene transcripts.

Gene	Accession number	Forward primer	Reverse primer
*CytB*	NC_012920 (14,747–15887)	CCACCCCATCCAACATCTCC	GCGTCTGGTGAGTAGTGCAT
*B2m*	NC_000015	TGTTCCTGCTGGGTAGCTCT	CCTCCATGATGCTGCTTACA
*ATF4*	NM_001675	GGCCAAGCACTTCAAACCTC	AGCAAGGAGGATGCCTTCTC
*Catalase*	NM_001752	CTCAGGTGCGGGCATTCTAT	AGCGGTCAAGAACTTCACTGA
*CHOP*	NM_001195053	CCTCCTGGAAATGAAGAGGAAGAA	GTCACAAGCACCTCCCAGAG
*GABPA1*	NM_002040	GGAACAGAACAGGAAACAATG	CTCATAGTTCATCGTAGGCTTA
*GPx1*	NM_000581	GGAGAACGCCAAGAACGAAG	CCAACTTCATGCTCTTCGAGAA
*GPx4*	NM_002085	AAGATCTGCGTGAACGGGG	CTGGGAAATGCCATCAAGTGG
*HSPA9*	NM_004134	AGGACGTGAGCAGCAGATTG	AGAAGACCGGCGAAAGAAGG
*LONP1*	NM_004793	AGGAGGTGAAAGCCCTGACT	AACCCCATCTACCTGAGCGA
*NQO1*	NM_000903	CTGGAGTCGGACCTCTATGC	GGGTCCTTCAGTTTACCTGTGAT
*NRF1*	NM_005011	TTGAGTCTAATCCATCTATCCG	TACTTACGCACCACATTCTC
*NRF2*	NM_006164	AACTACTCCCAGGTTGCCCA	AGCAATGAAGACTGGGCTCTC
*PMP70*	NM_002858	AGAATGGCGATGGCAAGAT	TCTCTTCACTGTGTCTCATAGGA
*p53*	NM.000546.6	GAAGAGAATCTCCGCAAGA	CGGATCTGAAGGGTGAAA
*Lamp1*	NM_005561	CAGATGTGTTAGTGGCACCCA	CATCCAGGCGTACCTTTCCAA
*TFB2m*	NM_022366	CCAAGGAAGGCGTCTAAGGC	CAAAGTGGTTGCGCTCGAAAG
*Tfam*	NM_003201	ATGGCGTTTCTCCGAAGCAT	TCAAGATGCTTATAGGGCGGA
*MRPL51*	NM_016497	TCTCTTGGTGTGCCTAGATTGA	CACTCCGAACATGGCCCTTTT
*HPRT1*	NM_000194	CCCTGGCGTCGTGATTAGTG	CGAGCAAGACGTTCAGTCCT
*C19orf74*	NM_001256440	ATGGAGGGGAAGTACGTCATC	GAGGCGGTCAAACACAGAC
*FAM57A*	NM_024792	TGAGCACTCCGTTTGTGTCG	CGGCCATAGGACCAGTACAT

### 2.16 Analysis of gene expression by RT-PCR

Total RNA was extracted with RNeasy Mini Kit (Qiagen, Düsseldorf, Germany), following manufacturer's protocols, and quantified using a Nanodrop2000 (ThermoScientific, Waltham, MA, USA), confirming that A260/280 was higher than 1.9. RNA integrity was verified by Experion RNA StdSens kit (Bio-Rad, Hercules, CA, USA), and RNA was converted into cDNA using the iScript cDNA synthesis kit (Bio-Rad), following the manufacturer's instructions. RT-PCR was performed using the SsoFast Eva Green Supermix, in a CFX96 real time-PCR system (Bio-Rad, Hercules, CA, USA), with the primers defined in Table 1, at 500 nM. Amplification of 25 ng was performed with an initial cycle of 30 s at 95 °C, followed by 40 cycles of 5 s at 95 °C plus 5 s at 60 or 63 °C. The amplification specificity was assessed at the end of the amplification by a melting curve between 65 and 95 °C, using an increment of 0.5 °C in each step. At the end of each cycle, Eva Green fluorescence was recorded to enable the determination of Cq. After amplification, the melting temperature of the PCR products was determined by performing melting curves, and amplicon length was confirmed by agarose electrophoresis. For each set of primers, amplification efficiency was assessed, and no template and no transcriptase controls were run. Gene expression was normalized to the geometric mean of RNA levels of 4 reference genes, including mitochondrial 37S ribosomal protein (MRPL51), hypoxanthine phosphoribosyltransferase 1 (HPRT1), C19orf74, and family with sequence similarity 57 member A (FAM57A) by using the CFX96 Manager software (v. 3.0; Bio-Rad).

### 2.17 Measurement of lysosomal proteolytic capacity

Lysosomal proteolytic capacity [53,54] was measured using the DQ Red BSA Dye (D12051, Thermo Fisher Scientific) following the manufacturer's protocol. Briefly, cells were seeded at a concentration of 15,000 cells/cm^2^ in white opaque-bottom, 96-well plates, with a final volume of 100 μl per well and allowed to proliferate for 24 h. Then, 25 μM of AntiOxCIN_4_ was added to cells for 48 h. In the last 24 h of the experiment, the cell culture medium was changed for OXPHOSm. Afterward, 1 mg of dye was resuspended in 1 mL of PBS 1x, and 100 μl of the resuspended dye was added to 10 ml of warm OXPHOSm. The cell culture medium was removed, and cells were loaded with 100 μl per well each of the dye-containing media and incubated at 37 °C for 1 h. Cells were then washed twice with warm PBS 1x, and the medium was replaced with 100 μL/well of warm EBSS medium (14155063, Thermo Scientific). The kinetic of DQ Red BSA digestion was monitored in a Biotek Cytation 3 spectrophotometer (BioTek Instruments Inc., USA) over a 4 h period at respective excitation and emission maxima of 495 nm and 525 nm.

### 2.18 Cell cycle analysis

Cells were seeded at a concentration of 15,000 cells/cm^2^ in tissue-culture dishes, with a final volume of 3 ml and allowed to proliferate for 24 h. Then, 25 μM of AntiOxCIN4 was added to cells for 48 h. In the last 24 h of the experiment, cell culture medium was changed for OXPHOSm. Afterward, cells were trypsinized, resuspended, and fixed with ice-cold (−20 °C) 70% ethanol for at least 24 h. Subsequently, cells were washed in PBST (PBS 1X, 0.1% Tween) and resuspended in 0.5 ml PBST containing 20 μg/ml RNase and incubated at 37 °C for 45 min. Propidium iodide (20 μg/ml) was added, and cells stained for 30 min at 37 °C. DNA quantity was measured using an Accuri C6 with an autosampler Flow cytometer (Becton Dickinson, USA). The percentage of cells in G1, S, and G2/M in each sample was determined using FlowJoWorkSpace 10.6.1 software (Becton Dickinson, USA).

### 2.19 Caspase-like activity assay

Cells were seeded at a concentration of 15,000 cells/cm^2^ in tissue-culture dishes, with a final volume of 10 ml and allowed to proliferate for 24 h. Then, 25 μM of AntiOxCIN_4_ was added to cells for 48 h. In the last 24 h of the experiment, cell culture medium was changed for OXPHOSm. Afterward, cells were trypsinized, and cellular pellets were diluted in caspase buffer assay (25 mM Hepes (pH 7.4), 10% sucrose, 10 mM DTT, 0.1% CHAPS). Then, 5 cycles of freeze-thaw liquid nitrogen were performed. The cellular extract was passed through 25G needle (25 strokes) and centrifuged at 14,000 rpm for 5 min. Then, the supernatant was collected, and protein concentration was determined by using the Bradford method and using BSA as a standard [50]. To measure caspase 3- and 9-like activity, aliquots of cell extracts containing 115 μg (for caspase 3) and 230 μg (for caspase 9) were incubated in a reaction buffer containing caspase buffer assay and 100 μM caspase substrate (Ac-DEVDpNA for caspase 3-like activity or Ac-LEHD-pNA for caspase 9-like activity (Calbiochem, Billerica, MA) for 2 h at 37 °C. Caspase-like activities were determined by following the detection of the chromophore p-nitroanilide after cleavage from the labeled substrate Ac-DEVD-p-nitroanilide or Ac-LEHD-p-nitroanilide. The method was calibrated with known concentrations of p-nitroanilide (Calbiochem). After incubation with caspase 3 and 9 substrates, absorbance was measured in a Biotek Cytation 3 spectrophotometer (BioTek Instruments Inc., USA) at 405 nm.

### 2.20 Catalase activity measurement

Catalase activity was determined by measuring hydrogen peroxide decomposition by following the 240 nm absorbance decrease [55]. Cells were seeded at a concentration of 15,000 cells/cm^2^ in tissue-culture dishes, with a final volume of 10 ml and allowed to proliferate for 24 h. Then, 25 μM of AntiOxCIN_4_ was added to cells for 48 h. In the last 24 h of the experiment, cell culture medium was changed for OXPHOSm. Afterward, cells extracts were resuspended in 50 mM Phosphate Buffer 50 mM, pH 7.8 (PB). Total cellular homogenate volumes equivalent to 80 μg of total protein were diluted with 200 μL PB in a multi-well plate. The catalase activity assay was started by the addition of 100 μL hydrogen peroxide solution at 10 mM. The 240 nm absorbance was read every 15 s for 3 min at 25 °C using a Biotek Cytation 3 spectrophotometer (BioTek Instruments Inc., USA). Purified catalase was used as a positive control. Separate wells containing the catalase inhibitor sodium azide were used as negative controls. For each sample, negative controls were prepared. The first seven absorbance readings of each sample and respective controls were fitted to an exponential regression curve. The maximal catalase activity was determined using the initial linear part of this fitting curve. Results are expressed in enzyme units (U) obtained directly from the decomposition of hydrogen peroxide using the Beer-Lambert law with l = 0.691 cm and Ɛ = 43.6 M^−1^ cm^−1^ [55].

### 2.21 Measurement of reduced and oxidized nicotinamide adenine dinucleotide phosphate levels

Reduced (NADPH), oxidized (NADP^+^), and NADPH/NADP^+^ ratio levels were determined by a NADP^+^/NADPH-Glo Assay kit (G9082, Promega, USA) following the manufacturer's instructions. Briefly, cells were seeded at a concentration of 15,000 cells/cm^2^ in tissue-culture dishes, with a final volume of 10 ml and allowed to proliferate for 24 h. Then, 25 μM of AntiOxCIN_4_ was added to cells for 48 h. In the last 24 h of the experiment, the cell culture medium was changed for OXPHOSm. Afterward, cell culture medium was removed and 50 μL of PBS 1x and 50 μL of 0.2 N NaOH with 1% DTAB were added to each well. After mixed in a plate shaker, 50 μL of each well was transferred to an empty well and 25 μl of 0.4 N HCl was added to each well. The plate was incubated for 15 min at 60 °C and then equilibrated for 10 min at room temperature. Twenty-five μl of 0.5 M Trizma base was added to wells containing acid-treated samples and 50 μl of HCl/Trizma solution was added to wells containing base-treated samples. Lastly, 100 μl of NADP/NADPH-Glo Detection Reagent was added and after 60 min of incubation at room temperature, the luminescence signal was monitored in a Biotek Cytation 3 spectrophotometer (BioTek Instruments Inc., USA).

### 2.22 Computational data analysis

Python 3 version 3.7.3 and Orange 3.27.1 were used for the computational data analysis. Pandas and SciPy packages were used to determine mutual information between each individual feature and the target (information gain). Cluster maps were plotted using Matplotlib and Seaborn modules to relate the features with higher mutual information (in columns) with instances (in rows), with the color of each cell representing the normalized level of a particular feature in a specific instance. The information is grouped both in rows and in columns by a two-way hierarchical clustering method using the squared Euclidean distance metric for both dendrograms.

To determine the capacity of the most informative features to predict the experimental class, we used Orange to apply principal component analysis (PCA) and plot a linear projection. As the features provided a good separation between the 4 experimental classes, we used the previous setup to train a supervised machine learning model, using support vector machines (SVM) with 5-fold cross-validation and evaluated its performance based on a confusion matrix, representing the number of true positives (TP), true negatives (TN), false positives (FP) and false negatives (FN). The following metrics were calculated: Precision = TP/(TP + FP); Recall = TP/(TP + FN); F1 score = 2 x ((Precision * Recall)/(Precision + Recall)), Classification Accuracy=(TP + TN)/(TP + TN + FP + FN) and Receiver Operating Characteristics (ROC) curve- FP/(FP + TN) vs Recall.

### 2.23 Statistical analysis

Data were analyzed using GraphPad Prism 6.01 (GraphPad Software, Inc. San Diego, CA, USA) and all results were expressed as mean ± standard error of the mean (SEM) for the number of experiments indicated in the legends of the figures. Statistical analysis was performed using one-way analysis of variance (ANOVA) followed by Dunnett's post hoc test for comparing effects of trichostatin and bafilomycin in fluorescent dyes used or two-way ANOVA followed by Turkey's multi-comparisons test for comparing effects of AntiOxCIN_4_. Values with p < 0.05 were considered statistically significant (*).

## 3 Results

### 3.1 AntiOxCIN_4_ restored mitochondrial membrane potential and mitochondrial fission of human skin fibroblasts from sPD patients

Initially, we evaluated AntiOxCIN_4_ cytotoxicity in fibroblasts from sPD patients and their sex- and age-matched controls, measured by the resazurin assay, to select a non-lethal AntiOxCIN_4_ concentration to be used in further experiments. Cells were treated with 1.5, 3, 6.25, 12.5, 25, 50 and 100 μM AntiOxCIN_4_ during 48 h (Fig. 1A). A decrease in metabolic activity was observed when fibroblasts from sPD patients and their sex- and age-matched controls were treated with 100 μM AntiOxCIN_4_ (Fig. 1B). Although a decrease in metabolic activity was not observed when cells were treated with 50 μM AntiOxCIN_4_, we observed a decrease in OCR, specifically in fibroblasts from healthy patients (Supplementary Figure 1A to C). Thus, all experiments were performed using 25 μM AntiOxCIN_4_. From this point on, all experiments were performed following the experimental design shown in Fig. 1A.

Considering that AntiOxCIN_4_ accumulation into mitochondria is dependent on Δψmt, we used vital epifluorescence imaging of cells loaded with the Δψmt-sensitive fluorescent dye TMRM to evaluate the effects of AntiOxCIN_4_ in mitochondrial network morphology and on Δψmt of fibroblasts from control and sPD patients. Our results demonstrated that fibroblasts from sPD patients have higher TMRM fluorescence (Fig. 1C and D). AntiOxCIN_4_ treatment restored TMRM fluorescence in fibroblasts from sPD patients to control values while not affecting control fibroblasts (Fig. 1C and D).

**Fig. 2:**
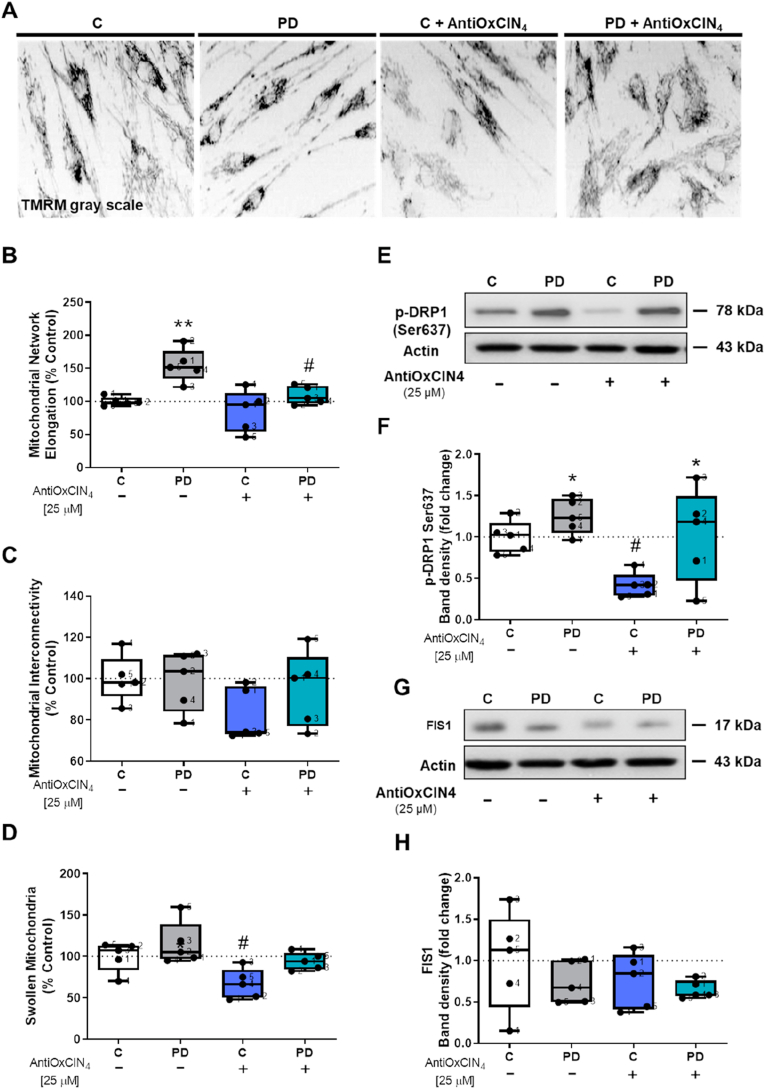
**Mitochondrial dynamics was restored by AntiOxCIN**_**4**_**treatment in fibroblasts from sPD patients.** Typical image of fibroblasts from sPD patients labeled with mitochondrial Δψ–dependent fluorescent probe TMRM (A). Mitochondrial network elongation (B), interconnectivity (C) and swelling (D) of these TMRM fluorescent cells were measured using an ImageJ macro (n = 45 cells per condition). Western blotting was used to detect phosphorylation of dynamin-related protein 1 at serine 637 (p-DRP1 ser637) (E and F) and mitochondrial fission 1 (FIS1) (G) protein contents in total fractions from human skin fibroblasts cell lines. Actin was used as a loading control. Blots are representative of different cell preparations with a random distribution between C and PD. Each measurement corresponds to one different individual (5 fibroblasts from sPD patients and 5 fibroblasts from respective sex- and age-matched healthy controls). Data was normalized by the control condition (C = 100% or C = 1.0 fold-change). Data are expressed as mean ± SEM of 5 different experiments. Statistical significance was accepted with (*) p < 0.05, (**) p < 0.01 to C vs PD or C + AntiOxCIN_4_ vs PD + AntiOxCIN_4_ and (^#^) p < 0.05 to C vs C + AntiOxCIN_4_ or PD vs PD + AntiOxCIN_4_.

Based on TMRM fluorescence, we next measured mitochondrial network elongation and interconnectivity, as well as swollen mitochondria using an ImageJ macro [49]. Our results showed more elongated mitochondria in fibroblasts from sPD patients, while AntiOxCIN_4_ treatment decreased mitochondrial elongation in the same cells (Fig. 2A and B). No differences were observed in mitochondrial interconnectivity (Fig. 2A and C). Regarding swollen mitochondria, we observed that AntiOxCIN_4_ treatment decreased swelling of mitochondria in fibroblasts from healthy controls (Fig. 2A and D).

Next, using western blotting, we semi-quantified proteins involved in mitochondrial fission, including p-DRP1 (ser637) and FIS1. Our results indicated that DRP1-Ser637-p was increased in fibroblasts from sPD patients (Fig. 2E and F), suggesting lower mitochondrial translocation. AntiOxCIN_4_ treatment decreased p-DRP1 (ser637) in fibroblasts from healthy controls but not from PD patients (Fig. 2E and F). However, no differences were observed in FIS1 protein content (Fig. 2G and H).

### 3.2 AntiOxCIN_4_ decreased autophagic fluxes in human skin fibroblasts from sPD patients

**Fig. 3:**
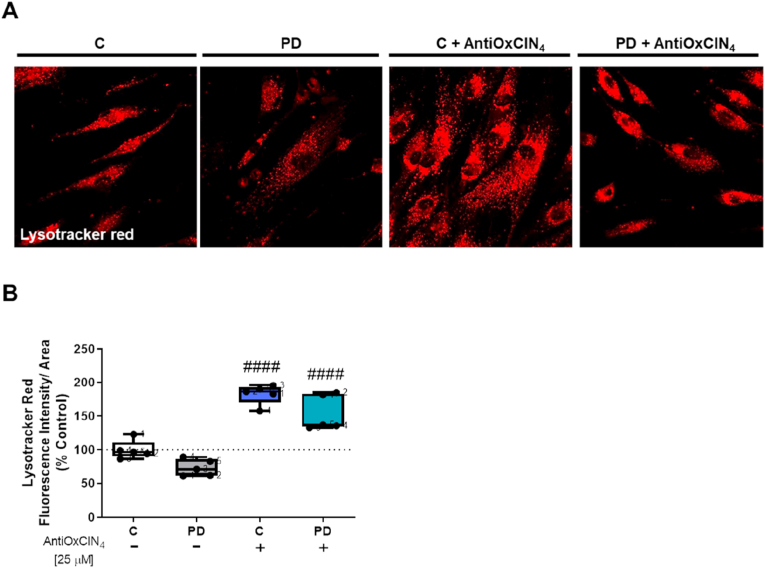
**AntiOxCIN**_**4**_**treatment of fibroblasts from sPD patients decreased Lysotracker red-labeled bodies.** A typical image of fibroblasts from sPD patients labeled with Lysotracker red in cells treated with vehicle (CT) or AntiOxCIN_4_ (A). Lysotracker red intensity fluorescence of human skin fibroblasts from sPD and their matched-controls was obtained by confocal microscopy and measured using FIJI/ImageJ 1.52p program (B). Each measurement corresponds to one different individual (5 fibroblasts from sPD patients and 5 fibroblasts from respective sex- and age-matched healthy controls). Data was normalized by the control condition (C = 100%). Data are expressed as mean ± SEM of 5 different experiments. Statistical significance was accepted with (^####^) p < 0.001 to C vs C + AntiOxCIN_4_ or PD vs PD + AntiOxCIN_4_. (For interpretation of the references to color in this figure legend, the reader is referred to the Web version of this article.)

Considering that mitochondrial malfunction can affect lysosomes and autophagy [86] and the mitochondrial morphological alterations observed in fibroblasts from sPD patients, we measured autophagy markers in order to evaluate the effects of AntiOxCIN_4_ in this pathway in both groups of cells. We first used Lysotracker Red, to label acid organelles, mostly lysosomes. Our results demonstrated that fibroblasts from sPD patients presented an apparent lower number of Lysotracker red-labeled bodies (Fig. 3A and B). Fibroblasts from sPD patients and their sex- and age-matched controls treated with AntiOxCIN_4_ showed an increased number of Lysotracker Red-labeled bodies (Fig. 3A–C). Trichostatin was used as a positive control, inducing autophagy [56], in which Lysotracker Red-labeled bodies were increased, while bafilomycin was used as a negative control by inhibiting autophagy [57]; in this case, no Lysotracker Red staining was obtained (Supplementary Fig. 2A and B).

**Fig. 4:**
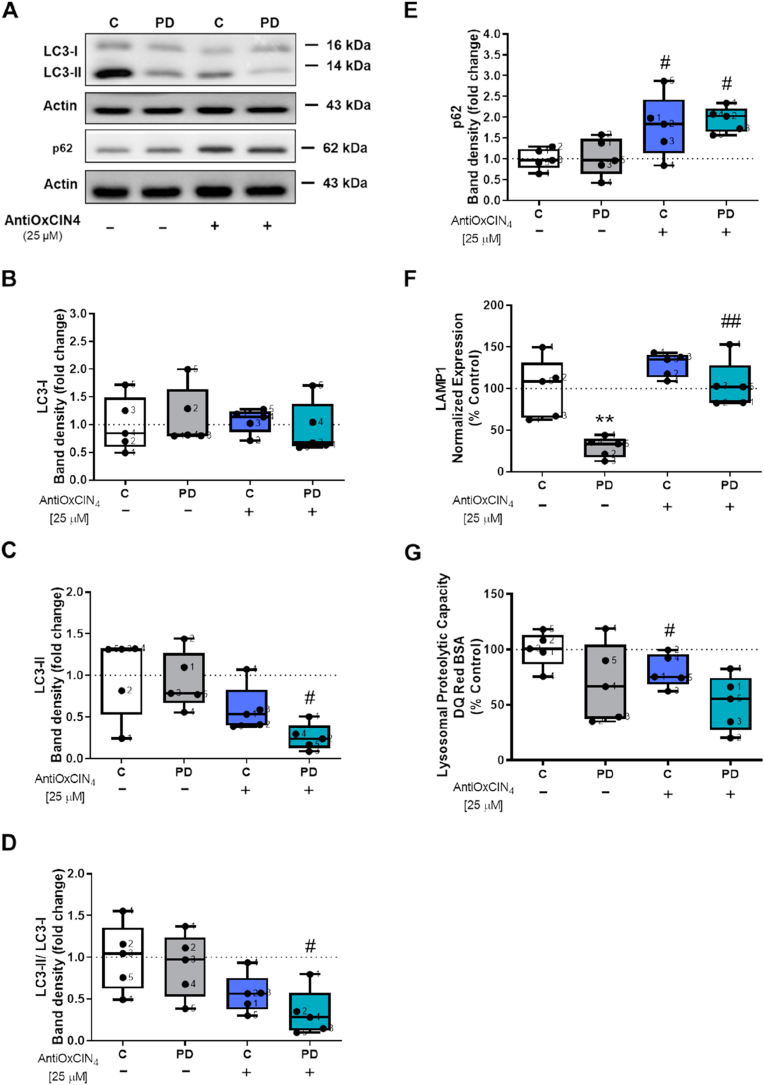
**Autophagic flux was decreased by AntiOxCIN**_**4**_**treatment of fibroblasts from sPD patients.**

Furthermore, we also measured several autophagy-related proteins content. We showed that LC3-II and LC3-II/LC3-I protein content ratio, an indicator of the autophagic flux, was decreased after AntiOxCIN_4_ treatment in fibroblasts from sPD patients (Fig. 4A, C and D), while no alterations were observed in LC3-I protein content (Fig. 4A and B). Additionally, an increase in p62 protein content was observed after treatment with AntiOxCIN_4_ in fibroblasts from sPD patients and their healthy controls (Fig. 4A and E). Regarding mRNA levels of lysosomal-associated membrane protein 1 (LAMP1), our results showed that fibroblasts from sPD patients presented decreased LAMP1 mRNA levels, while AntiOxCIN_4_ treatment restored that parameter to control values (Fig. 4F). AntiOxCIN_4_ treatment also decreased lysosomal proteolytic capacity in fibroblasts from healthy controls (Fig. 4G).

### 3.3 AntiOxCIN_4_ decreased mtDNA copy number and PGC1-α protein content in human skin fibroblasts from sPD patients

**Fig. 5:**
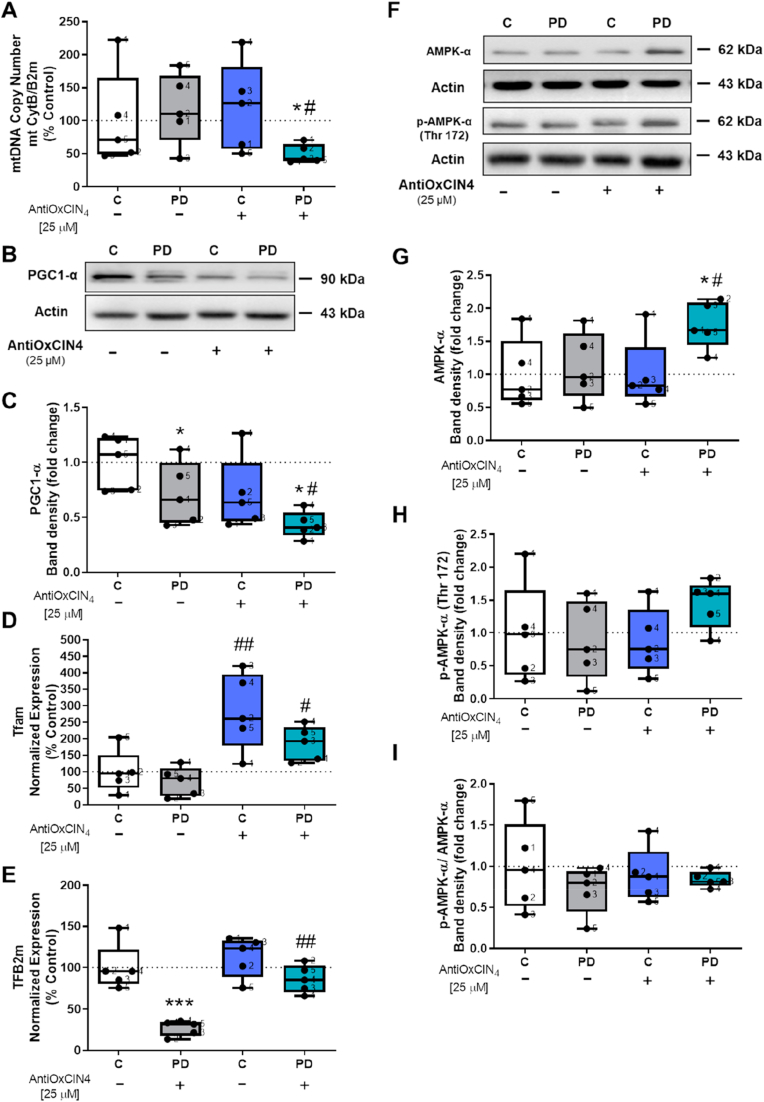
AntiOxCIN_4_ treatment of fibroblasts from sPD patients decreased mitochondrial biogenesis.

Next, we evaluated mitochondrial biogenesis by measuring mtDNA copy number, Tfam and TFB2m gene expression, and the protein content of PGC1-α, a master regulator of mitochondrial biogenesis. Furthermore, we measured protein amounts of several respiratory chain and ATP synthase subunits. mtDNA copy number (Fig. 5A) and PGC1-α protein content (Fig. 5B and C) were decreased in fibroblasts from sPD patients treated with AntiOxCIN_4_. However, AntiOxCIN_4_ treatment increased Tfam mRNA levels in both fibroblasts from sPD patients and in their sex- and age-matched controls (Fig. 5D), while TFB2m mRNA levels was increased only in fibroblasts from sPD patients (Fig. 5E). Fibroblasts from sPD patients presented decreased TFB2m mRNA levels compared with their control counterparts (Fig. 5E).

Furthermore, we also measured AMPK-α protein content using western blotting. Our results showed that fibroblasts from sPD patients treated with AntiOxCIN_4_ had increased AMPK-α protein content (Fig. 5F and G). However, no differences were observed in p-AMPK-α (thr172) protein content (Fig. 5F and H) and in the ratio between phosphorylated and total AMPK-α protein content (Fig. 5F and I).

We next semi-quantified the protein content of key mitochondrial respiratory chain-related subunits, including ATP synthase-related subunit (ATP5A), complex IV-related subunit (COX II) and complex II-related subunit (SDHB). TOM20, a central component of the import receptor complex that brings nuclearly encoded pre-proteins into mitochondria, usually used as an indicator of mitochondrial mass, was also semi-quantified. However, no alterations were observed in mitochondrial respiratory chain-related subunits protein content measured (Supplementary Figure 3A to D), as well as in TOM20 protein content (Supplementary Fig. 3E and F). AntiOxCIN_4_ treatment decreased SDHB protein content in control fibroblasts (Supplementary Fig. 3A and D).

The protein contents of sirtuin 1 (SIRT1) and 3 (SIRT3), which are NAD^+^-dependent type III histone deacetylases that regulate mitochondrial biogenesis and metabolism [58] were also determined. Nonetheless, no differences were found in SIRT1 (Supplementary Fig. 3G and H) and 3 (Supplementary Fig. 3G and I) protein content in any of the experimental groups.

### 3.4 AntiOxCIN_4_ increases maximal respiration and metabolic activity in human skin fibroblasts from sPD patients

**Fig. 6:**
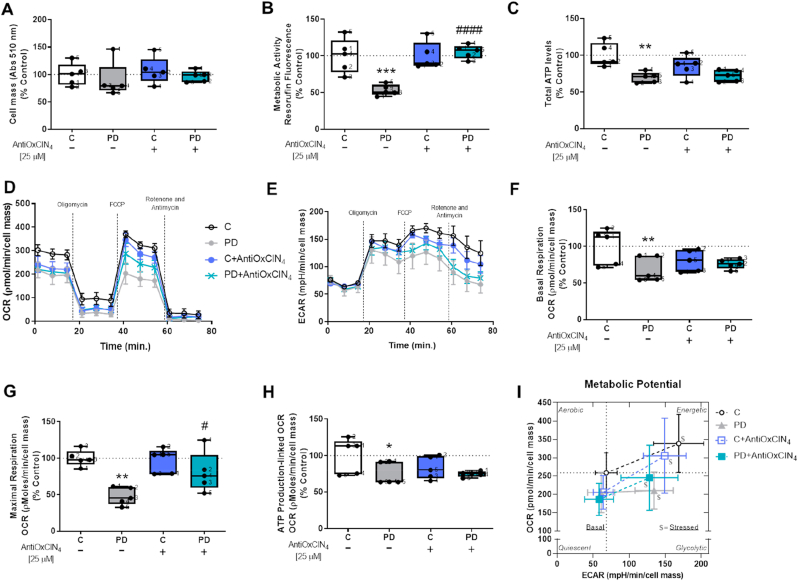
**Fibroblasts from sPD patients treated with AntiOxCIN**_**4**_**presented increased metabolic activity and mitochondrial maximal respiration.** Cellular proliferation (A) and metabolic activity (B) were measured using the sulforhodamine B (SRB) and resazurin assays, respectively. Intracellular ATP levels (C) were measured by using CellTiter-Glo Luminescent Cell Viability Assay (Promega) following manufacturer's instructions. The Seahorse XF^e^96 Extracellular Flux Analyzer was used to measure Oxygen Consumption Rate (OCR) (D) and extracellular acidification rate (ECAR) (E). Several OCR parameters were evaluated: basal cell respiration (F), maximal cell respiration (G) and ATP production-linked OCR (H) and different metabolic parameters (J). Energy map showing the metabolic potential of cells before adding of oligomycin (time point 3) and after being stressed with oligomycin plus FCCP (time point 7) (I). Each measurement corresponds to one different individual (5 fibroblasts from sPD patients and 5 fibroblasts from respective sex- and age-matched healthy controls). Data was normalized by the control condition (C = 100%). Data are expressed as mean ± SEM of 5 independent experiments. Statistical significance was accepted with (*) p < 0.05, (**) p < 0.01, (***) p < 0.005 to C vs PD or C + AntiOxCIN_4_ vs PD + AntiOxCIN_4_ and (^#^) p < 0.05, (^###^) p < 0.005 to C vs C + AntiOxCIN_4_ or PD vs PD + AntiOxCIN_4_.

To evaluate the effects of AntiOxCIN_4_ in mitochondrial function and metabolism, we measured cell mass, metabolic activity, total ATP levels, and rates of cellular metabolism using a Seahorse extracellular flux analyzer. As showed in Fig. 6, cell mass was not altered (Fig. 6A), while metabolic activity, measured as resazurin reduction, was decreased in fibroblasts from sPD patients, which was increased by AntiOxCIN_4_ treatment reaching values similar to the control (Fig. 6B). Fibroblasts from sPD patients had decreased total ATP levels (Fig. 6C), basal respiration (Fig. 6D and F), maximal respiration (Fig. 6D and G), ATP production-linked OCR (Fig. 6D and H) and basal ECAR (Fig. 6E and I). Treatment with AntiOxCIN_4_ increased maximal respiration in fibroblasts from sPD patients (Fig. 6D, G and J). No alterations were observed in other OCR-related parameters, including proton leak (Supplementary Figure 1D) and spare respiratory capacity (Supplementary Figure 1E). Under basal conditions, fibroblasts from sPD patients are less aerobic cells when comparing with their sex- and age-matched controls. Of note, metabolic stress, although increasing ECAR, caused only a small, non-significant increase in OCR. AntiOxCIN_4_ treatment of PD fibroblasts allowed stressed cells to increase OCR and ECAR (Fig. 6I).

### 3.5 AntiOxCIN_4_ induced S-phase cell cycle arrest in human skin fibroblasts from sPD patients

**Fig. 7:**
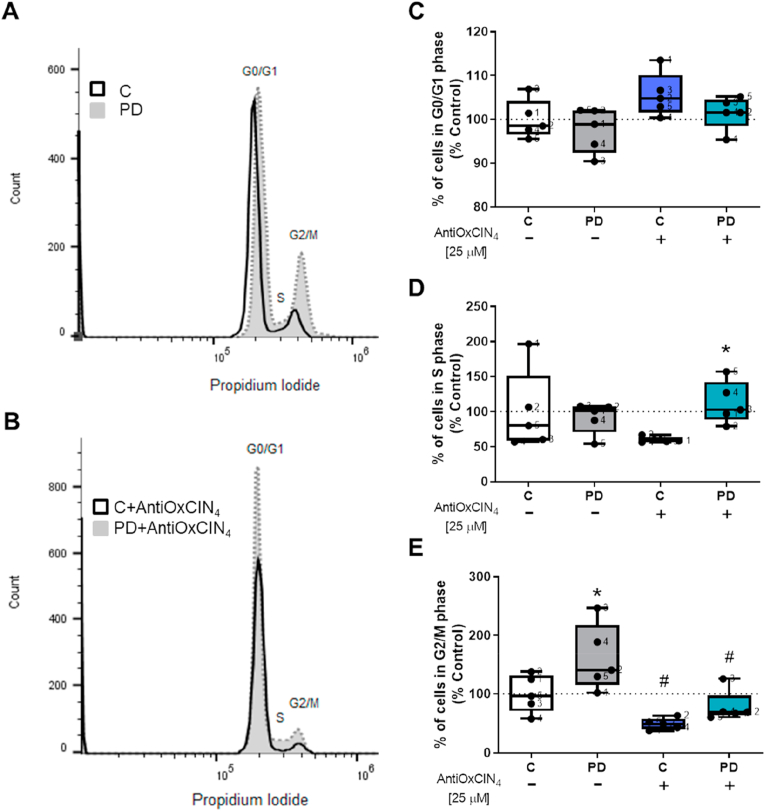
AntiOxCIN_4_ treatment of fibroblasts from sPD patients led to cell cycle arrest. Cell cycle analysis of human skin fibroblasts from sPD patients (PD) and their sex- and age-matched controls (C) were determined using flow cytometry. The percentage of cells in G1, S and G2/M in each sample was determined using FlowJoWorkSpace 10.6.1 software. Each measurement corresponds to one different individual (5 fibroblasts from sPD patients and 5 fibroblasts from respective sex- and age-matched healthy controls). Data was normalized by the control condition (C = 100%). Data are expressed as mean ± SEM of 5 different experiments. Statistical significance was accepted with (*) p < 0.05 to C vs PD or C + AntiOxCIN_4_ vs PD + AntiOxCIN_4_ and (^#^) p < 0.05 to C vs C + AntiOxCIN_4_ or PD vs PD + AntiOxCIN_4_.

Using flow cytometry, the effects of AntiOxCIN_4_ on the cell cycle were evaluated. Our results showed that fibroblasts from sPD patients had an increased percentage of cells in G2/M cell cycle phase (Fig. 7A and E), while AntiOxCIN_4_ treatment decreased the percentage of cells in that phase (Fig. 7B and E) and increased the percentage of cell in S cell cycle phase (Fig. 7B and D). Besides, fibroblasts from healthy controls treated with AntiOxCIN_4_ showed a decreased percentage of cells in G2/M phase (Fig. 7A and E). AntiOxCIN_4_ treatment did not change the percentage of cells in G0/G1 phase (Fig. 7A and C).

**Fig. 8:**
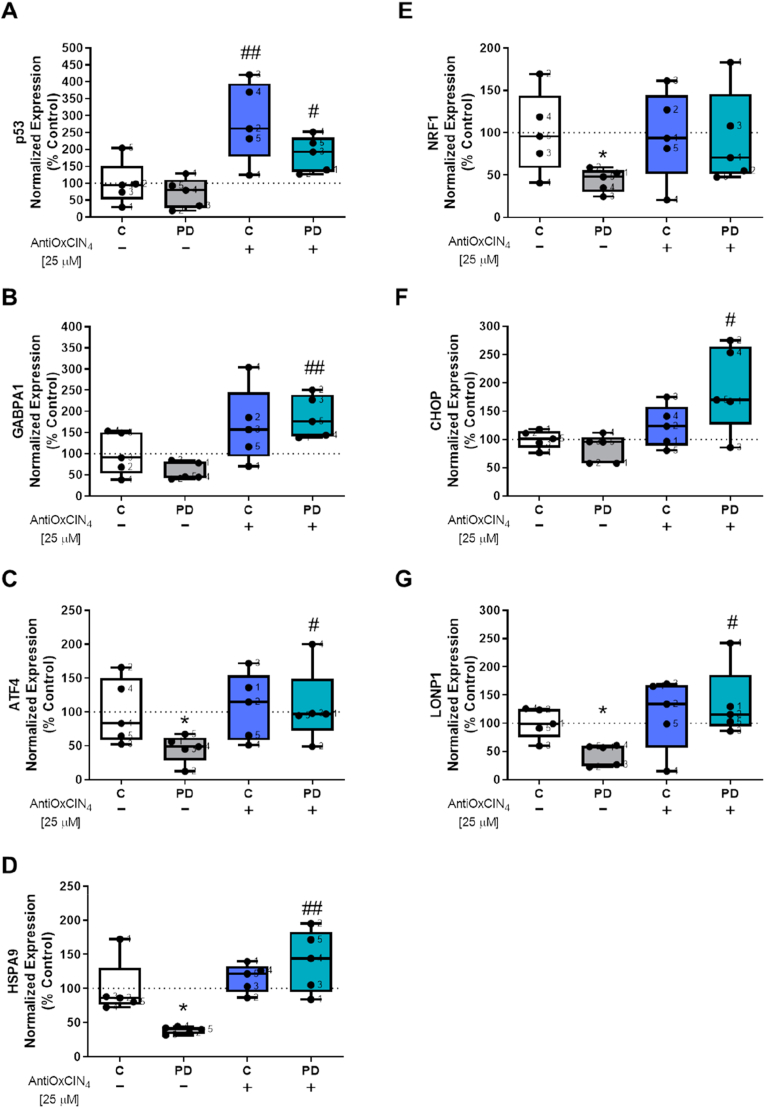
**AntiOxCIN**_**4**_**regulation of transcription factors related with cell cycle, cell survival, metabolism and mitochondrial function.** Total RNA was extracted, converted into cDNA, and amplified by real-time RT-PCR. mRNA levels of *p53* (A), *GA binding protein transcription factor subunit alpha 1* (*GABPA1*) (B), *activating transcription factor 4* (*ATF4*) (C), *heat shock protein family A (Hsp70) member 9* (*HSPA9*) (D), *nuclear respiratory factor 1* (*NRF1*) (E), *DNA damage-inducible transcript 3* (*CHOP*) (F) and *mitochondrial Lon peptidase 1* (*LONP1*) (G) were measured. mRNA levels were normalized to geometric mean of 4 housekeeping genes, including *mitochondrial 37S ribosomal protein* (*MRPL51*), *hypoxanthine phosphoribosyltransferase 1* (*HPRT1*), *C19orf74*, *family with sequence similarity 57 member A* (*FAM57A*). Each measurement corresponds to one different individual (5 fibroblasts from sPD patients and 5 fibroblasts from respective sex- and age-matched healthy controls). Data was normalized by the control condition (C = 100%). Data are expressed as mean ± SEM of 5 different experiments. Statistical significance was accepted with (*) p < 0.05 to C vs PD or C + AntiOxCIN_4_ vs PD + AntiOxCIN_4_ and (^#^) p < 0.05, (^##^) p < 0.01 to C vs C + AntiOxCIN_4_ or PD vs PD + AntiOxCIN_4_.

We also measured mRNA levels for several transcription factors related to the regulation of cell cycle and cell survival (*p53*), response to stress (*ATF4*, *CHOP*, *LONP1*, *HSPA9*, *HSP70*), metabolism, and mitochondrial function (*GABPA1, NFR1)* using RT-PCR. AntiOxCIN_4_ treatment increased *p5*3 mRNA levels in fibroblasts from healthy controls (Fig. 8A), while *GA binding protein transcription factor subunit alpha 1* (*GABPA1*) (Fig. 8B), *activating transcription factor 4* (*ATF4*) (Fig. 8C), *heat shock protein family A (Hsp70) member 9* (*HSPA9*) (Fig. 8D), *DNA damage-inducible transcript* 3 (*CHOP*) (Fig. 8F) and *mitochondrial Lon peptidase 1* (*LONP1*) (Fig. 8G) mRNA levels were increased in fibroblasts from sPD patients treated with AntiOxCIN_4_. Untreated fibroblasts from sPD patients had *ATF4* (Fig. 8C), *HSPA9* (Fig. 8D), *nuclear respiratory factor 1* (*NRF1*) (Fig. 8E), and *LONP1* (Fig. 8G) mRNA levels decreased.

### 3.6 AntiOxCIN_4_ improves cellular response to stress by increase cellular redox state and decrease ROS levels of human skin fibroblasts from sPD patients

**Fig. 9:**
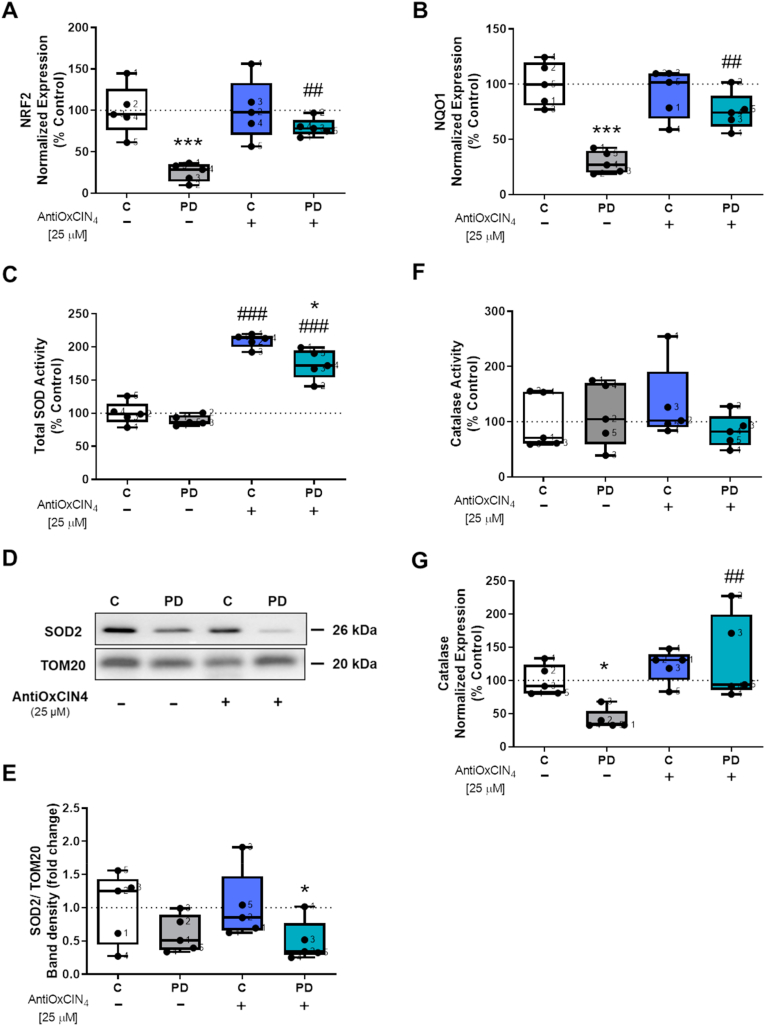
**AntiOxCIN**_**4**_**treatment of fibroblasts from sPD patients increased total SOD activity and upregulated NRF2 and NQO1 gene expressions.** Total RNA was extracted, converted into cDNA, and amplified by real-time RT-PCR. mRNA levels of *nuclear factor erythroid 2-related factor 2* (*NRF2*) (A), *NADPH quinone oxidoreductase-1* (*NQO1*) (B) and *catalase* (G) were measured. mRNA level was normalized to geometric mean of 4 housekeeping genes, including *mitochondrial 37S ribosomal protein* (*MRPL51*), *hypoxanthine phosphoribosyltransferase 1* (*HPRT1*), *C19orf74*, *family with sequence similarity 57 member A* (*FAM57A*). Total superoxide dismutase (SOD) activity (C) was measured using a commercially available kit, following the manufacturer's instructions. Western blotting was used to semi-quantify superoxide dismutase 2 (SOD2) in total fractions from human skin fibroblasts cell lines (D and E). TOM20 was used as a loading control. Blots are representative of different cell preparations with a random distribution between C and PD. Catalase activity was determined by following hydrogen peroxide decomposition by measuring the 240 nm absorbance decrease (F). Each measurement corresponds to one different individual (5 fibroblasts from sPD patients and 5 fibroblasts from respective sex- and age-matched healthy controls). Data was normalized by the control condition (C = 100% or C = 1.0 fold-change). Data are expressed as mean ± SEM of 5 different experiments. Statistically significance was accepted with (**) p < 0.05, (***) p < 0.005 to C vs PD or C + AntiOxCIN_4_ vs PD + AntiOxCIN_4_ and (^##^) p < 0.01, (^###^) p < 0.005 to C vs C + AntiOxCIN_4_ or PD vs PD + AntiOxCIN_4_.

AMPK-α activation increases cellular adaptative responses through erythroid 2 like 2 (NRF2) signaling [59]. To evaluate the effects of AntiOxCIN_4_ in the NRF2 pathway, we measured the mRNA levels of *NRF2* and *NADPH quinone oxidoreductase-1* (*NQO1*), a NRF2-target gene. We demonstrated that while fibroblasts from sPD patients had *NRF2* and *NQO1* mRNA levels decreased, treatment with AntiOxCIN_4_ increased its *NRF2* and *NQO1* mRNA levels (Fig. 9A and B, respectively). On the other hand, NRF2 signaling activation can trigger antioxidant pathways [60]. To investigate the effects of AntiOxCIN_4_ in modulating the antioxidant defense system and cellular responses to stress, we next measured some of the proteins involved in the antioxidant defense system, namely SOD catalase activities p66^shc^ pathway and the cellular redox state. Our results showed that treatment with AntiOxCIN4 increased total SOD activity in fibroblasts from sPD patients as well as their controls (Fig. 9C), while only fibroblasts from sPD patients treated with AntiOxCIN_4_ showed decreased levels of SOD2 protein content (Fig. 9D and E). Catalase activity was not altered (Fig. 9F). Although *catalase* mRNA levels were decreased in fibroblasts from sPD patients, treatment with AntiOxCIN_4_ increased *catalase* mRNA levels in the same cells (Fig. 9G).

**Fig. 10:**
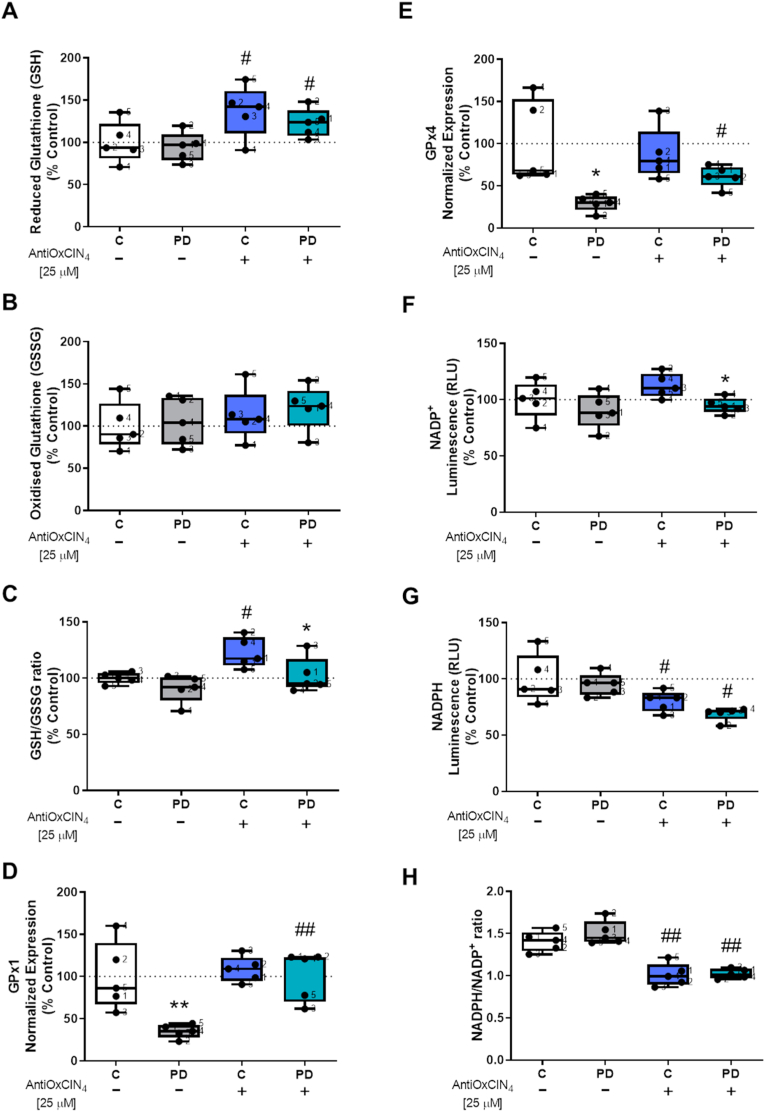
**AntiOxCIN**_**4**_**treatment of fibroblasts from sPD patients increased cellular redox state.** Reduced (A and G) and oxidized (B and F) forms of glutathione (GSH and GSSG, respectively) and nicotinamide adenine dinucleotide phosphate (NADPH and NADP^+^, respectively) and total cellular GSH/GSSG ratio (C) and NADPH/NADP^+^ ratio (H) levels were determined by a GSH/GSSG-Glo or NADP^+^/NADPH-Glo Assay kits, respectively, following manufacturer's instructions. mRNA levels of *glutathione peroxidase 1* (*GPx1*) (D) and *glutathione peroxidase 4* (*GPx4*) (E) were measured. mRNA level was normalized to the geometric mean of 4 housekeeping genes, including *mitochondrial 37S ribosomal protein* (*MRPL51*), *hypoxanthine phosphoribosyltransferase 1* (*HPRT1*), *C19orf74*, *family with sequence similarity 57 member A* (*FAM57A*). Each graphics measurement corresponds to one different individual (5 fibroblasts from sPD patients and 5 fibroblasts from respective sex- and age-matched healthy controls). Data was normalized by the control condition (C = 100%). Data are expressed as mean ± SEM of 5 different experiments. Statistically significance was accepted with (*) p < 0.05, (**) p < 0.01 to C vs PD or C + AntiOxCIN_4_ vs PD + AntiOxCIN_4_ and (^#^) p < 0.05, (^##^) p < 0.01 to C vs C + AntiOxCIN_4_ or PD vs PD + AntiOxCIN_4_.

To measure the effects of AntiOxCIN_4_ in cellular redox state, we next evaluated GSH and GSSG levels. We showed that fibroblasts from sPD patients and their sex- and age-matched controls treated with AntiOxCIN_4_ presented increased GSH levels (Fig. 10A), while no alterations were observed in GSSG levels (Fig. 10B). The ratio between GSH and GSSG levels was increased in control fibroblasts after treatment with AntiOxCIN_4_ (Fig. 10C). *Glutathione peroxidase 1* (*GPx1*) and *4* (*GPx4*) mRNA levels were both decreased in fibroblasts from sPD patients, while treated cells with AntiOxCIN_4_ increased *GPx1* and *GPx4* mRNA levels (Fig. 10D and E, respectively). Regarding NADP^+^ and NADPH levels, cofactors used in anabolic reactions and antioxidant response, our results showed that fibroblasts from sPD patients treated with AntiOxCIN_4_ had decreased NADP ^+^ levels (Fig. 10F). On the other hand, NADPH levels (Fig. 10G) and the ratio between NADPH and NADP^+^ levels decreased in both fibroblasts from sPD patients and in their sex age-matched controls treated with AntiOxCIN_4_ (Fig. 10G and H, respectively).

**Fig. 11:**
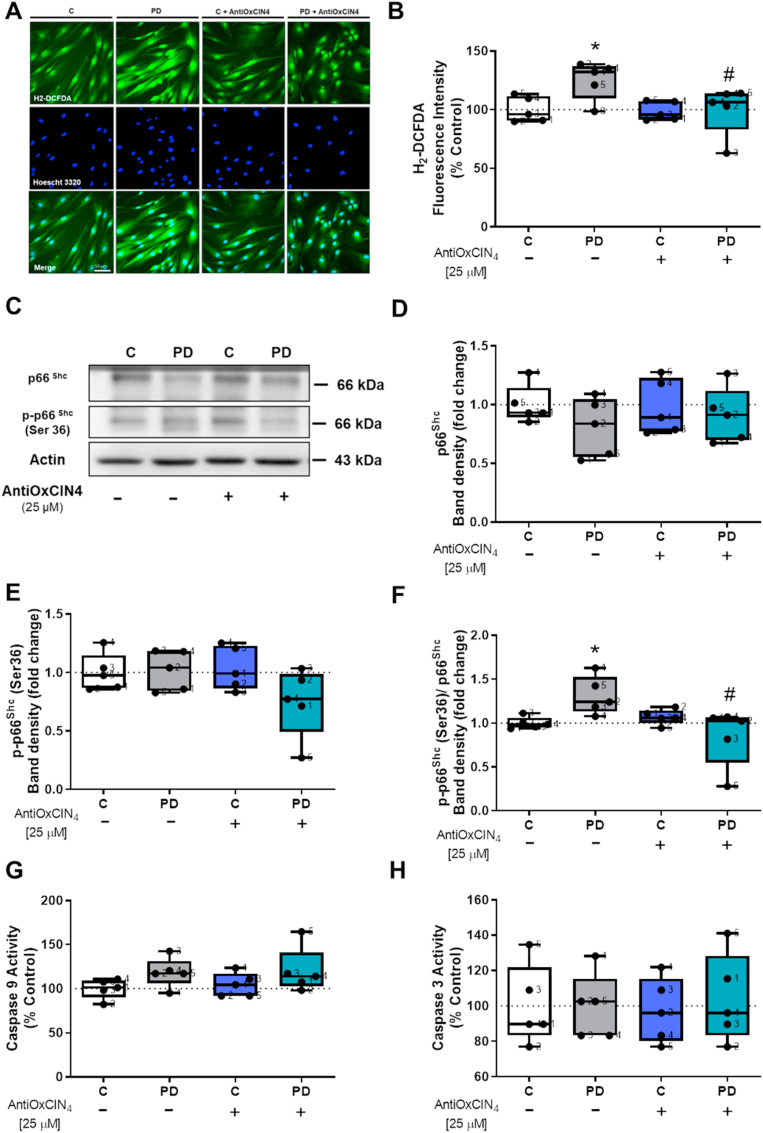
**AntiOxCIN**_**4**_**treatment of fibroblasts from sPD patients decreased H**_**2**_**-DCFDA oxidation.** H_2_-DCFDA intensity fluorescence of human skin fibroblasts from sPD and their matched-controls were measured by epifluorescence microscopy (A). The quantification of H_2_-DCFDA fluorescence was obtained by ImageJ 1.45S program. Graphic is expressed as mean ± SEM of H_2_-DCFDA intensity fluorescence divided by area (B). Western blotting was used to detect total p66^Shc^ (C and D), phosphorylation of p66^Shc^ (p-p66^Shc^ Ser36) (C and E) and the ratio between p-p66^Shc^ Ser36 and total p66^Shc^ (F) in total fractions from human skin fibroblasts cell lines. Actin was used as a loading control. Blots are representative of different cell preparations with a random distribution between C and PD. Caspase-9- (G) and caspase-3-like activities (H) were measured by the cleavage of the colorimetric substrates Ac-LEHD-pNA and Ac-DEVD-pNA, respectively. Caspase-like activity was expressed as the concentration of pNA released per μg protein. Known concentrations of p-NA were used as standards. Each measurement corresponds to one different individual (5 fibroblasts from sPD patients and 5 fibroblasts from respective sex- and age-matched healthy controls). Data was normalized on the control condition (C = 100% or C = 1.0 fold-change). Data are expressed as mean ± SEM of 5 independent experiments. Statistical significance was accepted with (*) p < 0.05 to C vs PD or C + AntiOxCIN_4_ vs PD + AntiOxCIN_4_ and (^#^) p < 0.05 to C vs C + AntiOxCIN_4_ or PD vs PD + AntiOxCIN_4_.

We also measured the p66^shc^ signaling pathway, which mediates oxidative stress-induced apoptosis [61]. Our results demonstrated that fibroblasts from sPD patients had increased CM-H_2_-DCFDA fluorescence intensity and treatment with AntiOxCIN_4_ decreased CM-H_2_-DCFDA fluorescence intensity in the same cells (Fig. 11A and B, and Supplementary Fig. 4A and B). Although no differences were observed in total p66^shc^ (Fig. 11C and D) and p-p66^shc^ (ser36) (Fig. 11C and E), we measured an increased p-p66^shc^ (ser36)/total p66^shc^ ratio in fibroblasts from sPD patients, while AntiOxCIN_4_ treatment decreased this ratio in the same cells (Fig. 11C and F).

### 3.7 AntiOxCIN_4_ did not trigger intrinsic apoptotic pathway in human skin fibroblasts from sPD patients

We next evaluated the intrinsic apoptotic pathway by measured caspase-9 and -3 activities. No differences were found in caspase-9-like (Fig. 11G) and caspase-3-like (Fig. 11H) activities. We also evaluated Bcl-2-associated X protein (BAX) protein content and B-cell lymphoma 2 (Bcl-2) by Western Blotting, since both have essential roles in the apoptotic pathway. However, no differences were found in BAX (Supplementary Fig. 4C and D) and Bcl-2 protein content (Supplementary Fig. 4E and F).

### 3.8 Total SOD activity, Lysotracker Red staining, mRNA levels of *HSPA9*, *LAMP1*, *TFB2m* and *NRF2*, and cells in G2/M phase provided a good separation between experimental groups

**Fig. 12:**
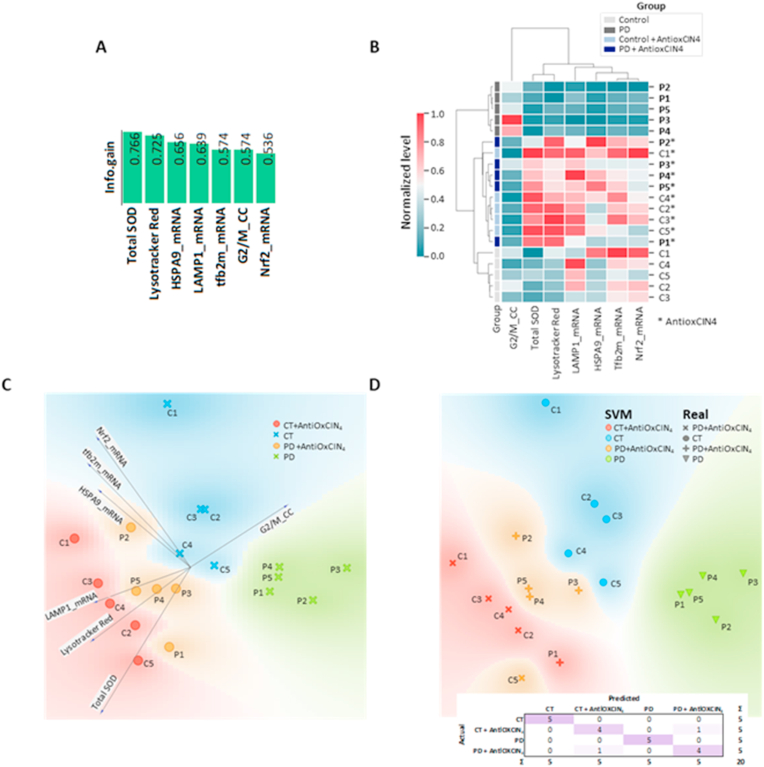
**Integrative data analysis.** A) Mutual Information (Information Gain) evidenced total SOD, Lysotracker Red staining, *HSPA9*, *LAMP1* and *TFB2m* transcripts, as well as G2/M phase (cell cycle) and *NRF2* transcripts as the features with more discriminative power between all experimental conditions. B) Selection of the 7 most discriminant features allowed a perfect separation of untreated control and PD samples into distinct clusters, while samples treated with AntiOxCIN_4_ were placed in the same cluster. C) PCA analysis using the same 7 features evidenced an excellent separation between the experimental conditions. D) Supervised machine learning analysis using support vector machines with cross-validation provided a Precision and Recall of 0.900 each, with an area under ROC curve of 0.887 and Classification Accuracy and F1 score of 0.900 each. Only two samples were misclassified (C5 + AntiOxCIN_4_ classified as PD + AntiOxCIN_4_ and PD1 + AntiOxCIN_4_ classified as C + AntiOxCIN_4_). (For interpretation of the references to color in this figure legend, the reader is referred to the Web version of this article.)

To understand which subset of features measured in this work contributed to better discrimination between the experimental groups, we determined the mutual information between each feature and the experimental class (C, PD, C + AntiOxCIN_4_, PD + AntiOxCIN_4_). A subset of 7 features returned an information gain higher than 0.5, containing total SOD activity, Lysotracker Red staining, mRNA levels of *HSPA9*, *LAMP1*, *TFB2m* and *NRF2*, and cells in G2/M phase (Fig. 12A). Hierarchical clustering using this subset of features provided perfect segregation of the PD samples. In the second level of the clustering, the control samples were segregated perfectly from the AntiOxCIN_4_-treated samples (Fig. 12B). Among the AntiOxCIN_4_-treated samples, the separation was not so evident. Then, we applied PCA using the same subset of 7 features and plotted a linear projection to examine each feature's importance in each experimental group, evidencing the similarities between samples from the same experimental group and the differences between different experimental groups (Fig. 12C). As the features provided a good separation, we trained an SVM model and evaluated its performance based on a confusion matrix (Fig. 12D), providing a Precision and Recall of 0.900 each, with an area under ROC curve of 0.887 and Classification Accuracy and F1 score of 0.900 each. Only two samples were misclassified (C5+AntiOxCIN_4_ classified as PD + AntiOxCIN_4_ and PD1+AntiOxCIN_4_ classified as C + AntiOxCIN_4_), again confirming that AntiOxCIN_4_ treatment brings PD cells closer to the phenotype found in the control group.

## 4 Discussion

**Fig. 13:**
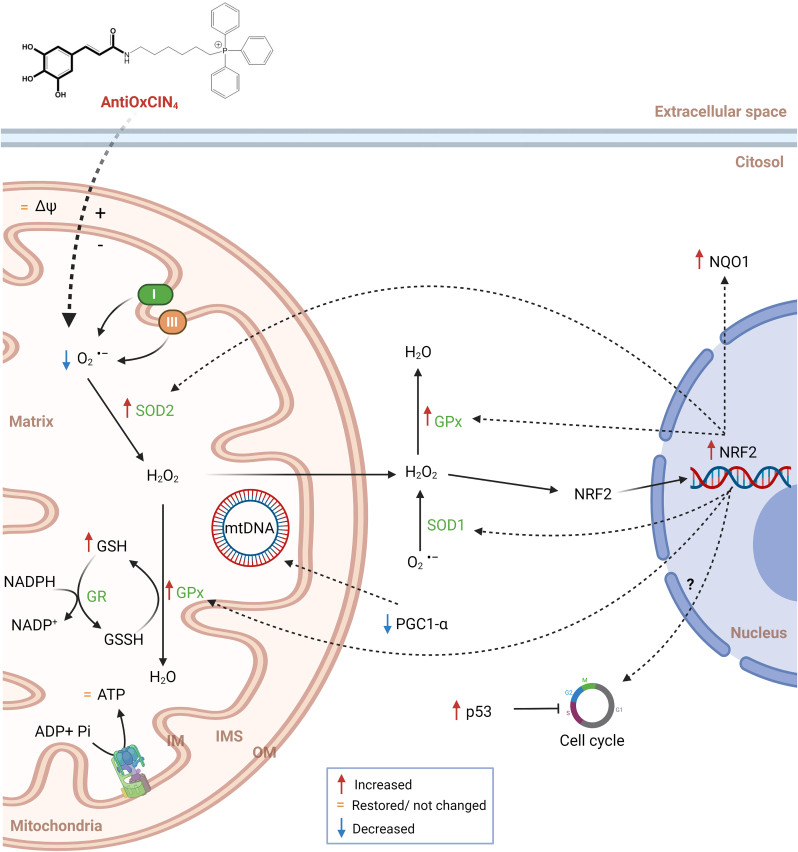
Schematic representation of the mechanisms underlying AntiOxCIN_4_ beneficial effects in fibroblasts from sPD patients. A new dietary antioxidant based on caffeic acid scaffold was conjugated with TPP cation (AntiOxCIN_4_), allowing its preferential accumulation into mitochondria. Treatment of fibroblasts from sPD patients with non-lethal AntiOxCIN_4_ concentrations restores mitochondrial membrane potential, decreases ROS levels and stimulates gene expression of the Nuclear factor erythroid 2-related factor 2 (NRF2). The latter stimulates the total activity of superoxide dismutases (SOD), thereby facilitating the conversion of mitochondrial superoxide (O_2_^•-^) into hydrogen peroxide (H_2_O_2_) and inhibiting ROS-induced cell death. Downstream of SOD action, H_2_O_2_ is converted into H_2_O by glutathione peroxidases (GPx) in a glutathione (GSH)-dependent manner. Oxidized GSH (GSSG) is regenerated to GSH by the action of glutathione reductase (GR), using electrons supplied by NADPH. AntiOxCIN_4_ increased the gene expression of the NAD(P)H dehydrogenase (quinone) 1 (NQO1), which is a NRF2 target. Due to low ATP levels of fibroblasts from sPD patients treated with AntiOxCIN_4_, AMPK promotes S-phase cell cycle arrest in a p53-dependent manner to restore normal mitochondrial function and avoid a deleterious mitochondrial phenotype. Activation of NRF2 can also result in cell cycle arrest at an early stage of oxidative stress response mechanisms. Downregulation of mitochondrial biogenesis and autophagic flux occurred as a compensatory mechanism and restored mitochondrial fission by decreasing mitochondrial elongation, most likely by decreasing DRP1 phosphorylation at ser673. A general improvement in mitochondrial health in fibroblasts from sPD patients is exhibited by restored maximal respiration, decreased mitochondrial swelling, and increased cellular metabolic activity (further details are provided in the Discussion).

We recently demonstrated that metabolic and specifically mitochondrial defects present in non-neuronal cells, such as fibroblasts from sPD patients, can be uncovered using a modified culture medium that stimulates mitochondrial ATP production [43]. Using the same strategy in this work, we showed that metabolic and mitochondrial alterations observed in fibroblasts from sPD patients could be recovered by a new mitochondriotropic dietary antioxidant, proposing a potential mechanism of action for the beneficial effects in mitochondrial function (Fig. 13). Furthermore, based on a supervised machine learning model using SVM, we demonstrated that AntiOxCIN_4_ treatment converted fibroblasts from sPD patients closer to their sex- and age-matched healthy controls (Fig. 12). Considering that fibroblasts can be collected from patients in a minimally-invasive manner, our work suggests that mitochondria-targeted antioxidants based on a polyphenol scaffold are a great promise in the prevention and/or treatment of PD, which can lead to significant savings in deterioration of patient health and shorten the time and cost for drug development. At the same time, this work validates the use of fibroblasts from sPD patients under forced mitochondrial oxidative phosphorylation as platforms for drug development since the correction of metabolic defects in peripheric cells may also be very significant in PD.

Currently, PD is an incurable disease in which pharmacological interventions can only control the symptoms. However, in more advanced stages of the disease, clinical treatment is no longer enough to control PD symptoms [2]. Some promising candidate neuroprotective agents are based on pathological and laboratory studies, but it has not been possible to find any drug with a disease-modifying effect in PD to date. Using strategies in a prodromal-PD phase, in which no motor symptoms are present, could be achieved by using peripheral tissues in which some biomarkers could be identified and/or the metabolic defects could be ameliorated by a proposed treatment. With this in mind, we hypothesized that using a mitochondria-targeted antioxidant (AntiOxCIN_4_), the metabolic phenotype found in PD can be improved. The accumulation of mitochondria-targeted compounds within the mitochondrial matrix is driven by the large ΔΨm, which is negative inside [62]. It is estimated that a hundred- or thousand-fold increase in the concentration of these compounds occurs inside mitochondria [62]. In fact, it was already showed that AntiOxCIN_4_ accumulation ratio in rat liver mitochondria is around 2000 fold and that sub-lethal concentrations did not alter nuclear morphology and mitochondrial polarization in HepG2 cell line [40], consistently with our results for healthy fibroblasts (Fig. 1C and D). However, fibroblasts from sPD patients showed hyperpolarized mitochondria, while AntiOxCIN_4_ treatment restored TMRM fluorescence intensity to values similar to healthy fibroblasts. Furthermore, AntiOxCIN_4_ treatment of fibroblasts from sPD patients restored mitochondrial fission by decreasing mitochondrial elongation, most likely by inhibiting DRP1 phosphorylation at ser673. Indeed mitochondrial fission/fusion machinery has an essential role in regulating cell cycle progression [63].

Numerous mitochondrial abnormalities have been extensively shown in several PD models [[6], [7], [8], [9]], including bioenergetics defects, alterations in mtDNA, generation of ROS, aberrant calcium homeostasis, abnormalities in mitochondrial dynamics and turnover, as well as impaired quality control mechanisms [64]. Mitochondrial dysfunction in PD leads to increased oxidative stress and/or vice versa, impacting several cellular signaling pathways [12]. Mitochondrial dysfunction and oxidative stress are present in asymptomatic stages of PD, and these alterations are not limited to the brain but also extend to peripheral tissues, as it is already established that PD is a multisystem disorder [16]. Some mitochondria-targeted antioxidants have demonstrated beneficial effects when tested in PD models [36]. MitoQ decreased mitochondrial fragmentation in 6-OHDA-treated SH-SY5Y cells, preventing the migration of DRP1 and BAX to mitochondria [65]. Moreover, in a 6-OHDA-induced *in vitro* and *in vivo* PD model, MitoQ protects dopaminergic neurons by enhancing Mfn2-dependent mitochondrial fusion through activation of PGC1-α [66]. Although some mitochondria-targeted antioxidants, namely MitoQ, SkQ1, MitoVitE, MitoTEMPO, MitoApocynin, MitoPBN and MitoSOD [36] showed beneficial effects against PD-associated oxidative stress, mitochondrial bioenergetics failure, and antioxidant defense system, a general overview about their cellular mechanism of actions in PD models is missing. Previously, we showed that AntiOxCIN_4_ has antioxidant and iron-chelation properties, inhibiting oxidative damage either in isolated liver mitochondria or in the HepG2 cell line [40]. Furthermore, neuroprotective effects were also shown in SH-SY5Y cells against 6-OHDA-induced oxidative damage [42]. Additionally, AntiOxCIN_4_ can increase GSH supply playing an important role in the maintenance of its intracellular levels [40]. Our group has recently demonstrated that AntiOxCIN_4_ (12.5 μM for 72h) increases ROS generation in primary human skin fibroblasts, stimulating a protective NRF2-dependent activation of antioxidant pathways, including by increasing SOD2 and GSH levels [41]. Concordantly, here we demonstrated that AntiOxCIN_4_ treatment of fibroblasts from sPD patients decreased ROS levels and cellular stress response, while metabolic activity, redox state and total SOD activity were increased, maximal respiration was restored, and mitochondrial swelling was decreased. Moreover, AntiOxCIN_4_ treatment of fibroblasts from sPD patients increased gene expression of several transcription factors that have important roles in regulating some signaling pathways, induced S-phase cell cycle arrest, and as a compensatory mechanism decreased auto(mito)phagy and mitochondrial biogenesis, while restoring mitochondrial dynamics (Fig. 13).

Although neurons are post-mitotic cells, cell cycle reactivation in mature neurons has been previously suggested to play a neurodegeneration role [67]. Furthermore, mitochondrial stress signaling can trigger cell cycle reactivation via E2F1, and a pro-apoptotic E2F1-dependent program in post-mitotic cells such as neurons (Raimundo etal,2012, Cell). Apoptotic cell death can occur following DNA damage. However, cell cycle arrest can occur before cell death initiation, allowing to repair the damage [68]. Evidence suggest a mitochondrial damage checkpoint (mitocheckpoint) correlated with S-phase cell cycle arrest [68]. Thus, when mitochondria are damaged or became dysfunctional, mitocheckpoint can be activated to help cells repair damaged mitochondria to restore normal mitochondrial function and avoid perpetuation of mitochondria-defective cells [69]. Mitocheckpoint can adjust cell cycle responses and gene expression to repair damaged mitochondria and restore normal mitochondrial function. Moreover, mitochondrial dysfunction leads to nuclear genome instability either by a ROS-dependent or ROS-independent pathways involved in mitochondria [70]. We observed a higher percentage of fibroblasts from sPD patients treated with AntiOxCIN_4_ in the S-phase of cell cycle, as well as a decrease in mtDNA copy number and protein content of PGC1-α, a master regulator of mitochondrial biogenesis, suggesting that cells are trying to repair mitochondrial abnormalities most likely avoiding the accumulation of defective mtDNA and mitochondria-defective cells. Furthermore, it is possible that a decrease in mitochondrial biogenesis and auto (mito)phagy observed in fibroblasts from sPD patients treated with AntiOxCIN_4_ is a compensatory mechanism due to mitochondrial damage checkpoint and cell cycle arrest, since defective mitochondria were decreased and, consequently, a decrease in quality control mechanisms occurred. Besides this, a decrease in autophagic flux observed in fibroblasts from sPD patients treated with AntiOxCIN_4_ was not accompanied by alteration in TOM20, a marker of mitochondrial content in the content of mitochondrial respiratory chain-related subunits. Moreover, changes in mitochondrial biogenesis induced by the AntiOxCIN_4_ seem to be caused by AMPK-α. Although AntiOxCIN_4_ treatment of fibroblasts from sPD patients resulted in increased Lysotracker Red-labeled bodies and *LAMP1* mRNA levels, meaning that lysosomal biogenesis is increased, it does not necessarily mean a more elevated autophagic capacity. It was previously shown that LAMP1 intensity did not represent degradative lysosomes or autolysosomes under physiological and pathological conditions in familial amyotrophic lateral sclerosis-linked motor neurons [71].

Furthermore, fibroblasts from sPD patients treated with AntiOxCIN_4_ showed increased *TFAM* and *TFB2m* mRNA levels, possibly as a compensatory mechanism in response to decreased mtDNA copy number and/or to a more efficient regulation of mtDNA structural stability, since TFAM and TFB2m determine the abundance of the mitochondrial genome by regulating packaging, stability, and replication [72,73].

In addition, p53 regulates several cellular processes: metabolism, autophagy, antioxidant response, DNA repair, cell cycle arrest, and apoptosis. Cell cycle arrest involving p53 is mediated by the transcriptional activation of p21/WAF1 [74]. Moreover, also ATF4 [75], GABPA1 [76], CHOP [77], LONP1 [78] can regulate cell cycle progression. Interestingly, transcripts of these genes were increased in fibroblasts from sPD patients after treatment with AntiOxCIN_4_. Furthermore, cell cycle and mitochondrial biogenesis are regulated by AMPK-α [79,80]. On the one hand, cell cycle regulation by AMPK-α is mediated by up-regulation of p53-p21 axis and regulation of mTORC pathway [79]. On the other hand, AMPK-α acts as an energy sensor of the cell and works as a key regulator of mitochondrial biogenesis [80]. Thus, AMPK-α mediates the cell cycle role as a metabolic checkpoint coordinating cell growth with energy status [81]. However, we show an increase in AMPK-α protein content while its activation shown by p-AMPK-α (thr172)/AMPK-α was not altered.

Besides, AMPK-α activation may boost cellular adaptative response and trigger antioxidant cascade through NRF2 signaling, a master regulator of anti-oxidative responses [59,60]. NRF2 regulates gene expression by binding to antioxidant responsive element (ARE), activating the expression of downstream genes such as NRF2-target genes are NADPH quinone oxidoreductase-1 (NQO-1), heme oxygenase-1 (HO-1), and glutathione S-transferase (GST). Thus, the NRF2 signaling activation is an adaptative response to the environmental and endogenous stresses [60]. In the same way, our results demonstrated that AntiOxCIN_4_ treatment of fibroblasts from sPD patients increased *NRF2* and *NQO1* mRNA levels. NRF2 can affect ROS and RNS levels by regulating the antioxidant systems through several mechanisms, including the induction of catabolism of superoxide and peroxides by SOD, Prx, and GPx, the regeneration of oxidized cofactors and proteins, and the synthesis of reducing factors. NRF2 knockout mice have a higher susceptibility to a broad range of chemical toxicity and disease conditions associated with oxidative pathology [82]. Furthermore, in the same study, pharmacological boosting of the NRF2 activity with chemoprotective agents protected animals from oxidative damage [82]. Moreover, at an early stage of the oxidative stress response mechanism, NRF2 may cause a transient cell cycle arrest by inhibiting Cyclin D1 [83].

Most of the mitochondria-targeted antioxidants tested so far in PD models have shown beneficial effects. For example, MitoQ scavenges peroxyl, peroxynitrite, and superoxide anion, protecting mitochondria against lipid peroxidation in SH-SY5Y cells treated with 6-OHDA [36]. Furthermore, MitoQ also stabilized mitochondrial morphology and function in 6-OHDA induced PD model, which further suppressed ROS formation and ameliorated mitochondrial fragmentation and cellular apoptosis [66]. We showed here an increase in total SOD activity, catalase, GPx1 and GPx4 gene expressions, as well as an increase in the mitochondrial redox state of fibroblasts from sPD patients after treatment with AntiOxCIN_4_, suggesting NRF2 regulation (Fig. 13). Glutathione reductase (GR) requires NADPH as a substrate to catalyzes the reduction of GSSG to GSH [84]. It was reported that although NADPH levels decreased, the glutathione levels can be increased either due to increasing de-novo synthesis of glutathione or reducing its usage [85], similarly with our observations. This suggests that AntiOxCIN_4_ restores GSH levels and consequently re-balanced the cellular redox state, using more NADPH. Subsequently, ROS production, cellular response to stress, and mitochondrial swelling were decreased, promoting an increased cellular metabolic activity and mitochondrial maximal respiration.

In conclusion, we demonstrated the beneficial effects of a new mitochondria-directed antioxidant in fibroblasts from sPD patients. Our mitochondria-targeted antioxidant can be considered a multi-functional compound since it has antioxidant capacity and regulates several signaling pathways that control mitochondrial metabolism and function. Hereby, we provide a possible mechanism of AntiOxCIN_4_ action (Fig. 13), leading to a deeper understanding of how that type of compounds can be used as a great promise in the prevention and treatment of PD, as well as the possibility to use skin fibroblasts from PD patients to identify cell proxies that can be used for an early diagnosis of PD in one approach of personalized medicine.

## 5 Contributions

CMD performed most of experiments, analyzed results, generated figures and wrote the manuscript. SPP, TCO, JT and RFS designed experiments and wrote part of the manuscript. FC, SB and FB synthetized the antioxidant compound used in the study. NR and PJO conceived and supervised the study, and wrote part of the manuscript. All authors critically revised the final form of the manuscript.

## Declaration of competing interest

The authors declare the following financial interests/personal relationships which may be considered as potential competing interests

PJO and FB are co-founders of the start-up MitoTAG, which had no role in funding or affecting this study outcomes. The funding agencies had no role in the decision to publish the manuscript.
