# Corrigendum to ‘A mitochondria-targeted caffeic acid derivative reverts cellular and mitochondrial defects in human skin fibroblasts from male sporadic Parkinson's disease patients’. [Redox Biology 45 (2021) 102037]

**DOI:** 10.1016/j.redox.2021.102117

**Published:** 2021-08-26

**Authors:** Cláudia M. Deus, Susana P. Pereira, Teresa Cunha-Oliveira, José Teixeira, Rui F. Simões, Fernando Cagide, Sofia Benfeito, Fernanda Borges, Nuno Raimundo, Paulo J. Oliveira

**Affiliations:** a PhD Programme in Experimental Biology and Biomedicine (PDBEB), Institute for Interdisciplinary Research (IIIUC), University of Coimbra, Coimbra, Portugal; b CNC-Center for Neuroscience and Cell Biology, CIBB - Centre for Innovative Biomedicine and Biotechnology, University of Coimbra, Coimbra, Portugal; c Research Centre in Physical Activity Health and Leisure (CIAFEL), Faculty of Sports, University of Porto, Porto, Portugal; d CIQUP/Department of Chemistry and Biochemistry, Faculty of Sciences, University of Porto, Porto, Portugal; e Penn State University College of Medicine, Department of Cellular and Molecular Physiology, Hershey, PA, USA; f Multidisciplinary Institute of Ageing (MIA), University of Coimbra, Coimbra, Portugal

2.3. Cell culture conditions

Skin fibroblasts from five sporadic late-onset PD (sPD) male patients and five age- and sex-matched healthy controls were obtained from a cell line repository of the Coriell Institute for Medical Research, USA (www.coriell.org), and their detailed information was previously described [43]. The repository ID numbers of control fibroblasts used were: ND29178, ND29179, ND34770, ND35044, and ND38530; the repository ID numbers of sPD fibroblasts used were: ND34265, ND35320, ND35322, ND35976, and ND39999.

