# Supplementary figures and images for: A mitochondria-targeted caffeic acid derivative reverts cellular and mitochondrial defects in human skin fibroblasts from male sporadic Parkinson's disease patients
Source: Redox Biol. 2021 Jun 8;45:102037. doi: 10.1016/j.redox.2021.102037 (PMC8220403; doi:10.1016/j.redox.2021.102037)

**Supplementary Figure 1**

**
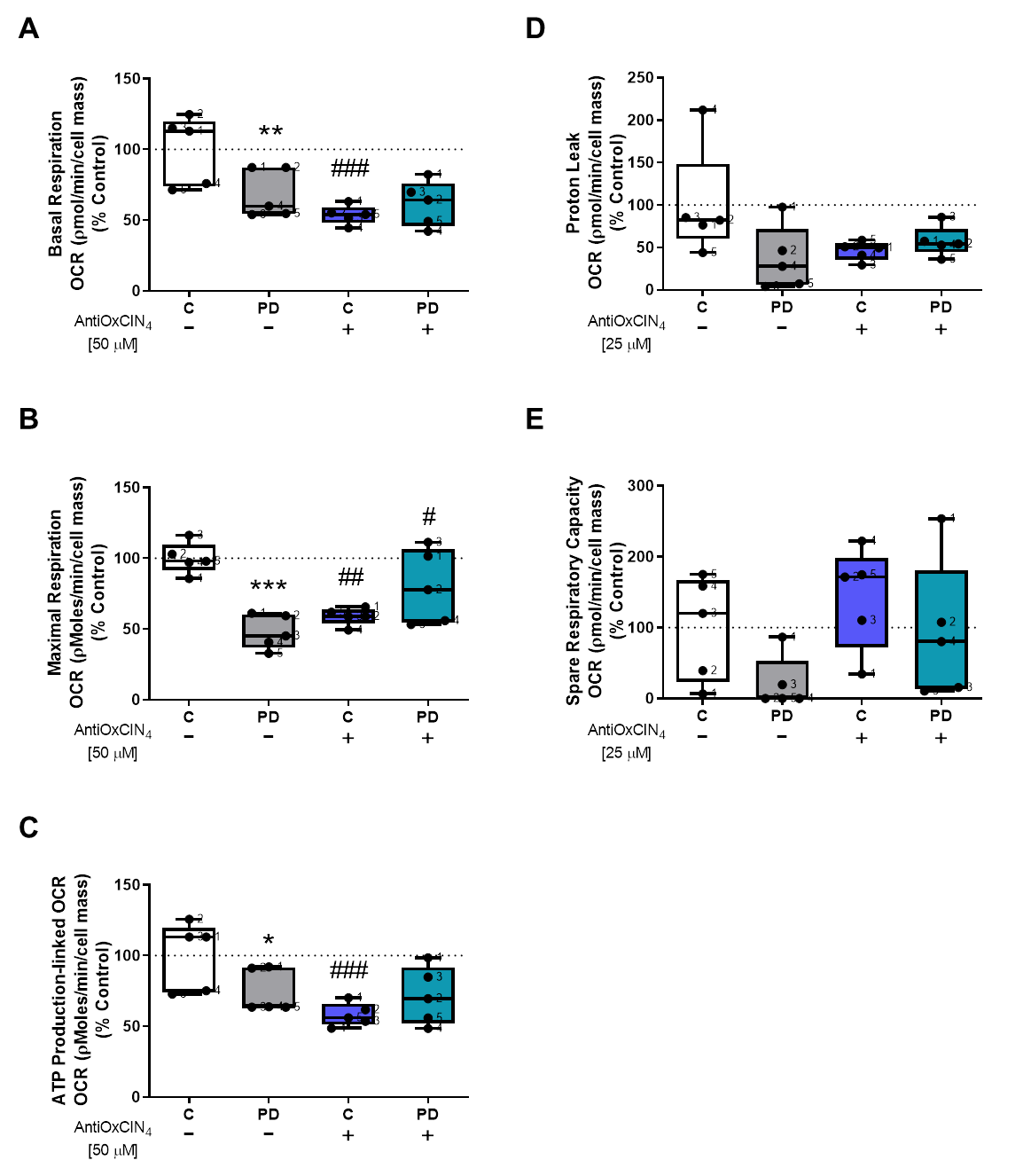
**

**Supplementary Figure 2**

**
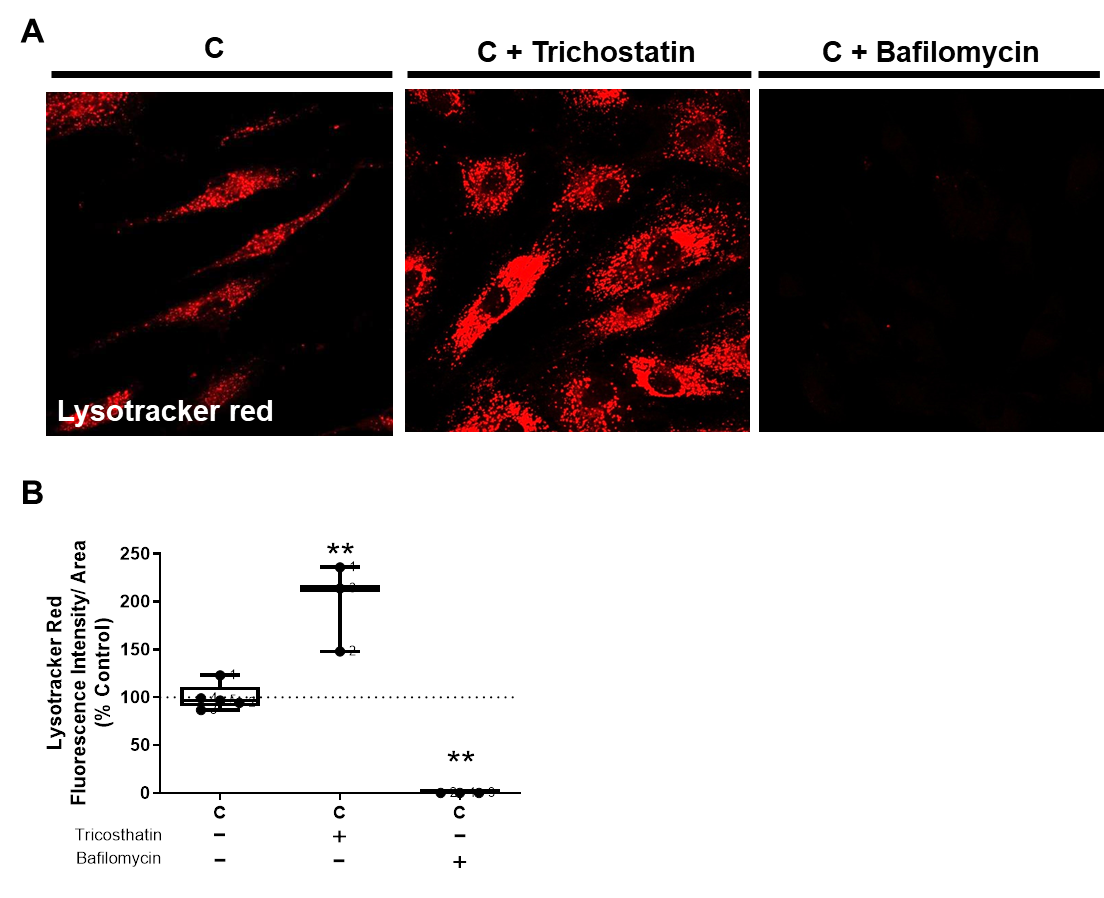
**

**Supplementary Figure 3**

**
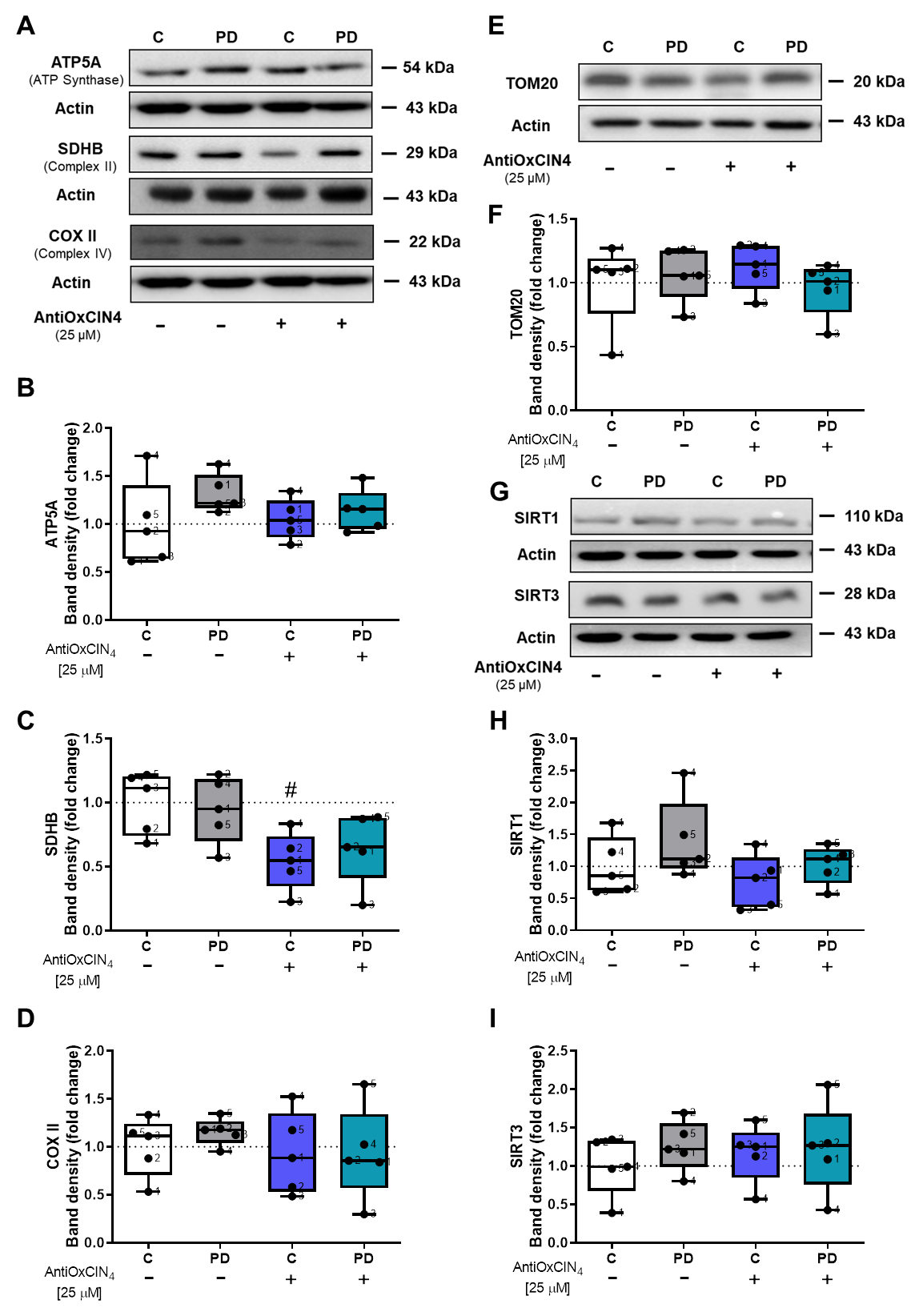
**

**Supplementary Figure 4**

**
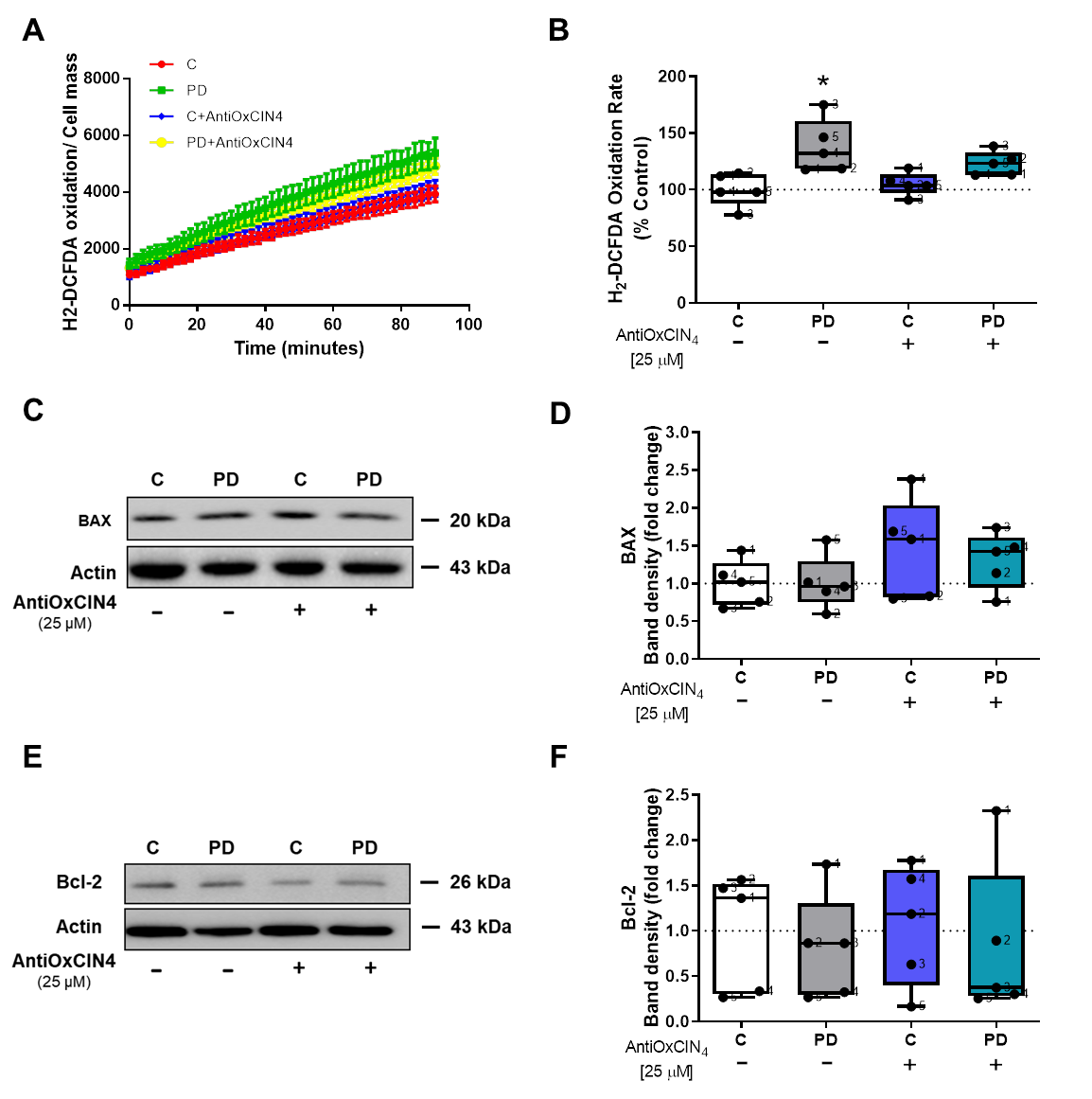
**

Supplement: Multimedia component 1 [file mmc1.docx]
